# Access to health services for chronic disease care during the COVID-19 pandemic in Ecuador: A qualitative analysis using a Social Determinants of Health approach

**DOI:** 10.1080/17441692.2023.2224859

**Published:** 2023-01-01

**Authors:** Marta Puig-García, María Fernanda Rivadeneira, Andrés Peralta, Elisa Chilet-Rosell, Ikram Benazizi-Dahbi, María Hernández-Enríquez, Ana Lucía Torres-Castillo, Cintia Caicedo-Montaño, Lucy Anne Parker

**Affiliations:** a Department of Public Health, Universidad Miguel Hernández (UMH), Alicante, Spain; b CIBER de Epidemiología y Salud Pública (CIBERESP), Madrid, Spain; c Institute of Public Health, Faculty of Medicine, Pontificia Universidad Católica del Ecuador (PUCE), Quito, Ecuador; d Centre of Community Epidemiology and Tropical Medicine (CECOMET), Esmeraldas, Ecuador

**Keywords:** Diabetes mellitus, hypertension, social determinants of health, healthcare disparities, COVID-19

## Abstract

This qualitative study aims to explore how the COVID-19 pandemic impacted healthcare access for patients with chronic conditions in Ecuador from the patient’s perspective. We interviewed 19 patients diagnosed with arterial hypertension or type 2 diabetes in rural and urban areas of Ecuador during August and September 2020. We used the Framework Method to analyse the interview transcripts with ATLAS.Ti 8.4 and organised the ideas discussed using categories from the World Health Organization Commission on the Social Determinants of Health conceptual framework. Reorganization of health services during the pandemic meant that patients with arterial hypertension or diabetes could no longer attend face-to-face appointments for disease follow-up. System failures related to medication supply led to increased out-of-pocket payments, which, together with reduced or absent earnings, and in a context with limited social protection policies, meant that patients frequently went for prolonged periods without medication. Rural health initiatives, support from family and use of traditional medicine were reported as ways to manage their chronic condition during this time. Barriers to disease management disproportionately affected individuals with low socioeconomic positions. Stock shortages, lack of protective labour policies and limited reach of anticipatory policies for health emergencies likely worsened pre-existing health inequities in Ecuador.

## Introduction

In the wake of the COVID-19 pandemic, the lack of health workers, personal protective equipment and hospital beds has strained the capacity of health systems around the world.(Chiriboga et al., 2020) The situation is especially worrying in low- and middle-income countries due to the historically weakened health systems. Debilitated and under-resourced health systems quickly became overwhelmed with the management of the pandemic, resulting in a greater impact on the most vulnerable populations.(Chiriboga et al., 2020; Katsidzira et al., 2020; Menendez et al., 2020; Nott, 2020) In Ecuador, where this study is focussed, the health system is fragmented and significant out-of-pocket health costs, even before the pandemic, have been described.(Lucio et al., 2011; Peñafiel-Chang et al., 2020)

It is widely reported that by focusing all efforts on controlling the pandemic curve, health care for other pathologies decreased and patient follow-up was set aside.(Banach et al., 2020; COVIDSurg Collaborative, 2020; World Health Organization, 2020c) According to the World Health Organization (WHO) approximately half of the countries surveyed had partially or completely disrupted services for hypertension treatment and for diabetes and diabetes-related complications.(World Health Organization, 2020b) In many cases, consultations were conducted remotely by telephone. Both diabetes and hypertension disproportionately affect people of low socioeconomic status, and they encounter greater difficulties in disease management due to a lack of resources, support networks, housing space and income.(Williams et al., 2018) It is likely that this limited interaction with face-to-face health services has generated additional challenges for self-management and following preventative advice.(Banerjee et al., 2020) Experts predict that when COVID-19 infections are under control, there will be a second wave of patients with urgent pathologies neglected during the pandemic.(Holmes et al., 2020; Meneu et al., 2020) Time will gradually unveil the severity of the interruption of care for chronic patients.(Remuzzi & Remuzzi, 2020)

The issue is made more complicated because many individuals with chronic health conditions are considered high-risk for COVID-19. Having diabetes increases the mortality of the virus (Huang et al., 2020) and diabetes patients facing a challenging socioeconomic environment are more vulnerable to COVID-19.(Basu, 2020) Furthermore, the very people who are most likely to be infected are also more likely to suffer chronic health conditions. Some authors propose that we are facing a syndemic where the virus clusters with pre-existing conditions, such as poverty, political instability, noncommunicable diseases, and lack of access to health services (Horton, 2020). Moreover, the social determinants of health modulate the intensity of the COVID-19 impact. For example, impacts related to lost or reduced employment have particularly affected women, given that measures to restrict mobility and social distancing have led to the cessation of the highly feminised service sector.(Castellanos-Torres et al., 2020)

In this way, COVID-19 and its containment measures may have exacerbated prior health inequities, creating new vulnerabilities for people with lower socioeconomic positions. The unequal socioeconomic impacts of COVID-19 and non-COVID-19-related health inequities are intertwined, (World Health Organization, 2020a) as is the case of non-communicable diseases (NCD).(World Health Organization, 2020b) Effectively addressing the emergent nature of the impacts of the COVID-19 pandemic on non-COVID healthcare access and on health equity will require a closer look at the experiences and views of the people directly involved or affected. This study aims to examine how the COVID-19 pandemic has impacted healthcare access for patients with diabetes and hypertension in Ecuador from the patient’s perspective.

## Materials and methods

### Study design

Qualitative study with a health equity approach in which we conducted semi-structured interviews during the COVID-19 pandemic with adult patients diagnosed with arterial hypertension or diabetes in Ecuador.

### Participants

Between 17th August and 17th September 2020, we conducted telephone interviews with people who have diabetes and/or hypertension. In-person interviews were not feasible due to the movement restrictions introduced to combat COVID-19. Recruitment used purposive sampling seeking to include a group of people with diabetes and/or hypertension that was heterogeneous in terms of gender, age and socioeconomic status in two very different areas of Ecuador. In Quito, the nation’s capital, we recruited individuals in low-income districts of the city, with the help of the patient clubs for people with diabetes at different public primary care facilities (Chimbacalle, La Magdalena, Historic Centre and Comite del Pueblo). These sectors are characterised by being traditional neighbourhoods of the capital, with high population density, high internal migration and a significant percentage of unsatisfied basic needs. Furthermore, through the Centre for Community Epidemiology and Tropical Medicine’s (CECOMET) network of lay health promoters, we recruited individuals from rural communities in Eloy Alfaro, Esmeraldas. Dense forest, limited infrastructure, and a majority afro-Ecuadorian population characterise this rural area on the northern coast of Ecuador. It is important to consider that people living in rural communities in Ecuador, especially Afro-Ecuadorian and indigenous populations, face significant economic, political and social inequalities. These communities suffer marginalisation and exclusion from the country’s economic and productive system, which limits their access to resources and opportunities for development. These inequalities are reflected in the lack of access to basic educational, health, recreational and technological services, as well as in the limited supply of adequate employment and consumption opportunities.(Sánchez, 2013) We increased the number of participants until we reached saturation and no new information was obtained.

### Setting and epidemiological context

#### Epidemiological context

The first confirmed case of COVID-19 in Ecuador was registered on 29th February 2020 in the city of Guayaquil. Ecuador was the third country in Latin America to confirm COVID-19 cases, after Brazil and Mexico. As a result, a health emergency was declared in the country on 11th March 2020. Cases of COVID-19 spread rapidly throughout all provinces (administrative-territorial units) of the country, making it one of the Latin American countries most affected by the pandemic. During the interview data collection period, cases of COVID-19-related disease had already spread to all provinces and the case fatality rate stood at 6.8% of confirmed cases.(Johns Hopkins Coronavirus Resource Center, 2020) On the 17th of March, the government imposed a full lockdown, limited transport, and ordered a curfew which at times endured from 2 pm to 5 am. General confinement obliged the population to stay at home except for purchasing food or medication. A ‘humanitarian law’ was implemented on 19th June 2020 which allowed both public and private companies to reduce the dedication and salaries of their employees by 50% (art. 20) and prevented redundancies unless the company was declared bankrupt. It also guaranteed stability of health workers (art. 25) and the creation of new positions for frontline workers.(Registro Oficial del Ecuador, 2020)

#### Ecuador’s health system

The health system in Ecuador is characterised by its segmentation and fragmentation, determined by the coexistence of subsystems with different modalities of financing, affiliation and provision.(- Molina Guzmán, 2019) Thus, public services of the Health Ministry and the social security system coexist with private services. The lack of a single system hinders, among other things, adequate follow-up of individuals and uniformity in access to care, as well as limiting the continuity of information to ensure quality and efficiency. Furthermore, the health system is divided into three levels of care, ranging from a lower level of specialisation to a more complex one. While in Quito these three levels coexist, in the rural area of Esmeraldas considered here only the first two levels are available, and access to health specialities is limited. Moreover, in isolated communities, medical doctors work consecutively for 22 days and take 8 days off, leaving rural communities without continuous access to primary care clinicians.

### Data collection and analysis

We conducted semi-structured, in-depth telephone interviews. We developed and piloted an interview topic guide to conduct the interviews, which consisted of open-ended questions focused on three aspects: experience of the disease prior to the pandemic, the management of the disease during the pandemic and personal experiences during the COVID-19 lockdown (supplementary file S1). Interviews were carried out by a team of four (1 male and 3 female) people with a health-related undergraduate degree who received prior training in interview techniques and were contracted specifically for the purpose of conducting and transcribing the interviews. At the beginning of each interview, we collected data including age, disease history, location, education, marital status, occupation and monthly income. After completing each interview, the interviewers recorded detailed field notes describing any issues they felt relevant pertaining to the interview (e.g. frequent interruptions, perceived presence of a third-party prompting interviewee’s responses). The duration of the interviews ranged from 16 to 53 min.

The interviews were audio-recorded, transcribed verbatim in Spanish, anonymised and uploaded to ATLAS.Ti 8.4 for thematic content analysis. Tone of voice, pauses and interruptions were documented during the interviews and described in the transcriptions by the interview team. We used the Framework Method to qualitatively analyse the interview data in order to compare and contrast data by categories across the different interviews while keeping the context of each perspective and individual’s experience.(Gale et al., 2013) After an in-depth reading of the interviews, seven members of the research team (MPG, MFR, AP, ECR, LAP, IBD, ALT) agreed on a basic set of codes for an initial analysis, in line with the structure of the interview guide. After this first analysis, we discussed the suitability of the codes created and adapted them for a second analysis. With this iterative approach, each interview was independently coded by at least 2 people. A proposed set of codes was shared and discussed with the interview team and the researchers charged with coordinating recruitment (MHE, CCM). We made a comparison of the codes used and discussed possible discrepancies and saturation.

We identified categories by crossing the coded information and classifying it according to the WHO Commission on the Social Determinants of Health (CSDH) conceptual framework as (a) Intermediary Determinants of Health and (b) Socioeconomic and Political Determinants of Health. Among the structural determinants of health and equity in the CSDH framework there are factors that generate social hierarchies and define differential access or vulnerabilities according to one’s socioeconomic position.(World Health Organization, 2010) To integrate this part of the framework into our analysis, three researchers (MPG, MFR, LAP) independently compared the different codes among subgroups according to the socioeconomic position (gender, migration status, education, occupation, income and location) and explored the perceived impact on equity. We analysed the interviews in Spanish and, only after analysis, the selected extracts to be included in the manuscript were translated into English by members of the research team (MPG, MHE, LAP), (two Spanish native speakers; one from Spain, another from Ecuador and one English native speaker). The original language version of the extracts can be accessed in supplementary file S2.

## Results

Twenty participants completed the interview; however, one individual from the rural setting was later excluded because it was felt that the responses to the questions given were highly influenced by the presence of her spouse. Of the remaining 19 participants, nine were women and the median age was 56 years (range 43−73 years) (Table 1). Thirteen (68.4%) participants lived in Quito and six (31.6%) lived in rural areas of Esmeraldas (Eloy Alfaro). Seventeen had a diagnosis of type 2 diabetes, and five of arterial hypertension (three individuals had both conditions). More than half of the participants were married. Overall, the participants were of relatively low socio-economic status, with more than half of the interviewees, mostly from Quito, having income below $400, the Basic Monthly Salary (BMS). Four participants had achieved tertiary education and the different occupations held included being a street vendor, small-scale agriculture, cleaner or taxi driver. Two participants were unemployed.

**Figure 1:**
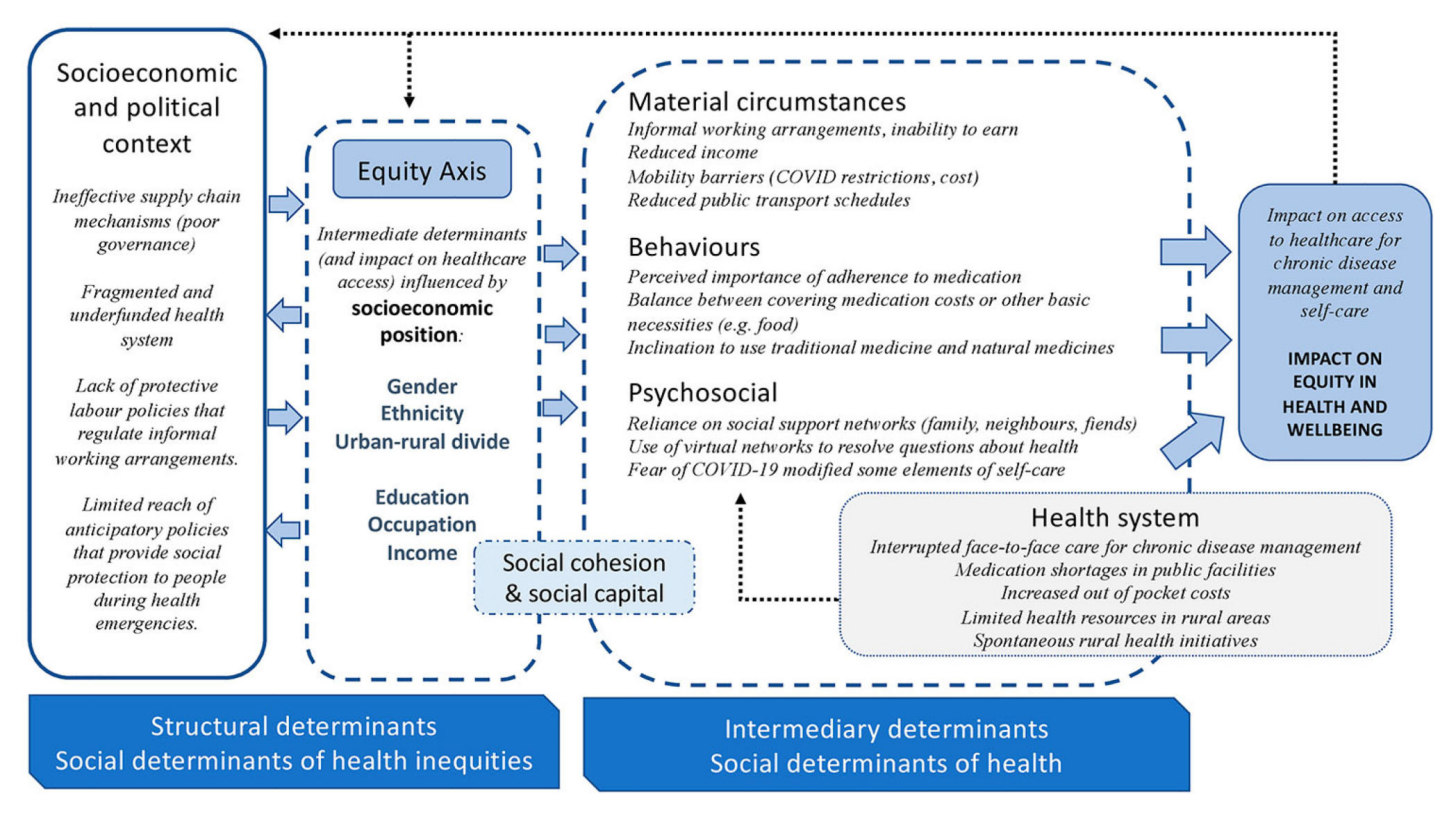
Adapted WHO CSDH conceptual framework showing how the impact of the pandemic on healthcare access for diabetes and hypertension in Ecuador is modulated by the social determinants of health and equity.

In an attempt to address the complex and interlinked nature of the factors that influence access to healthcare, we summarised and organised the converging discourses of the participants according to the WHO Framework of Social Determinants of Health (Figure 1).

### Intermediary determinants of healthcare access

#### Health system

During the pandemic, the collapse of primary care and hospitals with COVID-19 patients forced the healthcare system to reorganise. Health centres would no longer be open to receive routine patients face-to-face and the only way to get a medical appointment in Quito was through the call centres. Several participants described difficulties with accessing non-COVID care through the call centre.

Look, I call the call centre and they don’t want to give me an appointment, so now I have to go for some tests on the 20th [August] and I don’t know how I will go because the call centre won’t give me an appointment. − Female, 55, street vendor, income below BMS (Quito)

When routine appointments were cancelled, patients were still able to retrieve their medication from the pharmacies located within their healthcare facilities. However, most of the participants reported shortages in medicine stock in care centres and hospitals, and difficulties reaching the facilities due to the limited mobility. Consequently they had to purchase their medication in private pharmacies, increasing their out-of-pocket costs.

January, February, March, until March they gave me medication, from March until now [August] I have been buying it because there is no way to get down there [to the health centres] … nor are there any medicines in the hospitals or health centres. − Male, 70, trader, income below BMS (Quito)

Limited resources for health in rural areas make managing chronic diseases more challenging even without the restrictions imposed by the COVID-19 pandemic. Participants discussed the lack of qualified staff in rural health facilities and interrupted access when facilities closed to allow the health workers to visit isolated communities. They also mentioned the lack of basic material to manage chronic diseases, such as glucometers or batteries.

In the countryside, here, we are without, without all the medical specialities that can attend to us, for example: I mean a psychologist to help us with emotional support, or permanent doctors, one doctor leaves and another comes … if there is only one doctor, he goes out to the communities to visit, then the sub-centre is closed and there are no consultations for several days. − Female, 43, teacher, income above BMS (Esmeraldas)

Several interviewees from Esmeraldas described health workers visiting their residence during the lockdown period to facilitate the medication and check-ups.

Yes, with the doctors I had …, I had a medical appointment at home, (…) so they came and checked us and gave us some medicine that they brought with them. Before, we used to go out, but because of the pandemic, we couldn’t go out at all. − Male, 58, lab assistant, income above BMS (Esmeraldas)

Others described how doctors adjusted their medication prescription to diminish the need to access the health centre.

They have been giving me [medication] all the time, every time I go up and sometimes during these times the doctor would even prescribe my medication for two months so as not to run the risk of going up to the subcentre. − Female, 43, teacher, income above BMS (Esmeraldas)

## Material circumstances

Participants described how informal employment shaped their access to health services during the pandemic. People with unregulated employment, such as street vendors, reported being greatly affected by the lockdown due to their inability to work and losing their only source of income.

Right now, there is no work, the only work is to go out and sell in the street and we can’t do that − Female, 50, street vendor, income below BMS (Quito)

Reduced income was frequently cited as a reason for treatment interruption because individuals were unable to purchase their medication when it was unavailable in the public health system.

Imagine 3 months locked up, 3 months without work, without being able to do anything (…) I take the pills when I can buy them and sometimes, I’m late. I take them for 8 days, I stop them for 8 days and so on. (…) they send us to the hospitals, but there isn’t any [medication] at the hospitals …− Male, 59, construction worker, income below BMS (Quito)

Economic difficulties also impacted healthcare access and treatment adherence in other ways by creating mobility barriers, especially in rural settings. Both men and women in Esmeraldas discussed the high transport costs and the increased price of gasoline during the pandemic.

Because of the pandemic it [the price of canoe fares] went up, some charge $7, others charge $5 … it was as high as $8 at the beginning. − Female, 51, teacher, income above BMS (Esmeraldas)

Furthermore, reduced public transport schedules during the pandemic made accessing health services more difficult, ultimately having a negative impact on their health.

There was no transport, there was no mobility, so I couldn’t go out, and that has affected me, and lately I’ve been feeling bad, and I’m feeling a bit bad right now, and this week I went to the doctor because I was able to go out, I went to the doctor and what is the surprise …, my blood pressure has shot up, and my blood sugar is extremely high − Male, 61, farmer, income below BMS (Esmeraldas)

Regarding working conditions, the impact of the pandemic was not always negative and had the potential to remove access barriers. Some individuals, particularly men, mentioned how, before the pandemic, work schedules were not compatible with healthcare demands.

No, I didn’t visit [the health centre] before the pandemic because, I mean, because I didn’t have the time, thank God I had a job, I had a full work schedule. Going to the health centre means spending 3 to 4 hours and at work they don’t allow me that kind of time. − Male, 50, construction worker, income below BMS (Quito)

### Behaviours

Regarding adherence to medication and controls, in general, individuals were aware of the importance of being adherent and expressed a will to take medication, although this was hindered by limited access to the facilities and medication shortages during the pandemic. Some participants reported discontinuing their treatment when they were unable to cover the costs, despite being aware of the potential impact on their health.

So, I sometimes buy [medication] when I can, when I can’t, I don’t! And so, when I take it, as I say, I get better, but when I stop taking it, I … I start getting worse. − Male, 59, construction worker, income below BMS (Quito)

Others reported continuing treatment even if it meant limiting covering other basic needs.

Can you usually afford to buy medicines or other products necessary for your health care? Answer: Of course. For health you have to stop eating rather than stop taking pills, because … don’t you see? … when you are healthy, you can work. − Male, 61, farmer, income below BMS (Esmeraldas)

Traditional medicine use was widely reported, not only as a regular behaviour based on ancestral knowledge, but also as a measure to overcome the lack of access to medication for financial reasons, both before and during COVID-19 pandemic.

There have been times when I haven’t even had enough money, not even to buy a pill, but you have to find your own way here with … with home-made treatment, medicines, emmm … vegetables. − Female, 51, teacher, income above BMS (Esmeraldas)

Frankly, I have not [been able to access the health centre] ‘mi señorita’, I have been taking care of myself at home with my diabetes medication, honestly with natural medicine. − Female, 52, unemployed, income below BMS (Quito)

This use of natural remedies was frequently described by women, particularly those from the rural setting in Esmeraldas, where participants reported that they were able to manage their condition thanks to the availability of traditional medicines grown in their gardening plots. All educational groups discussed the use of natural remedies, although individuals with higher education expressed its use in combination with the prescribed medication.

Now I am, I am taking guava leaf, which they tell me controls sugar and I am taking that, today, every day. I boil my pot of water and add guava leaf and I am taking that every day; besides my pills. − Female, 51, teacher, income above BMS (Esmeraldas)

### Psychosocial factors

A person’s social support network appeared to play a major role in mediating healthcare access during the pandemic. Due to financial or access constraints, participants, especially women, frequently mentioned that they relied on their family members, friends, or neighbours to obtain their medication.

Those who are taking care of us are my children, they are giving us all the food, medicine and fruit, they are taking care of me at the moment. − Female, 52, unemployed, income below BMS (Quito)

The availability of family support may be mediated by socio-economic status, with more educated or wealthy participants more likely to have contacts who can help them negotiate solutions to their access problems (social capital).

I … got the medicine from a, from a, from a … cousin who works, lives in Esmeraldas. And she, through the doctors, she would go with my ID card to some doctors, and she would get the medicine and send it to me, because I’ve been unable to see my doctor for a long time. − Female, 51, teacher, income above BMS (Esmeraldas)

For some, the availability of family support was impacted by the pandemic as family members themselves dealt with challenging socio-economic conditions.

I have a son who used to buy my pills but … now … they also cut his salary; he works at C [private company]. So, he used to buy my pills and he got them for me … but now with the situation they cut his salary, they took away his overtime, everything (…) he no longer has enough for himself, because he also has expenses. − Male, 59, construction worker, income below BMS (Quito)

While participants from Quito less frequently described the importance of relying on friends and family to help them overcome barriers to access during the pandemic, they did describe health centres with telephone chat groups to resolve questions and uncertainties around management of the disease. Participants reported that this was helpful when they could not access control appointments with the doctor.

I only [went to the health centre] when I had a medical appointment (…). Since then, I haven’t gone at all, not at all, not at all, as we have a group chat [WhatsApp group] here in the health centre for the diabetic patient’s club. We have also consulted there any concerns. − Male, 53, taxi driver, income below BMS (Quito)

Additionally, under psychosocial factors, we can consider how emotions such as fear influenced access to diabetes and hypertension care during the pandemic. Participants that reported having lost a family member to COVID-19 tended to be wary of leaving their homes or communities to retrieve or purchase medication. Furthermore, perceived vulnerability to COVID-19 (due to diabetes or being elderly) prevented people from approaching health centres.

For safety reasons I am no longer going to the health centre because of this situation. You know that for safety reasons, entering a hospital is a bit … risky (…) we are vulnerable to …, diabetics are vulnerable in this situation. − Male, 65, printing service worker, income below BMS (Quito)

### Structural determinants of inequity in healthcare access

We have integrated reflections about how certain socio-economic factors, such as one’s employment status, gender, educational status, or residence in a rural setting, interact with the intermediate determinants of healthcare access described above (Figure 1). Additionally, one interviewee reported perceived discrimination by medical doctors due to her immigration status and the need to share her medication with her husband.

My husband is without pills for both diabetes and hypertension, so we are both taking the pills they gave me for me, that’s why I’m not taking the complete medication. He doesn’t have anything for his hypertension. He’s dying with a headache every day because of hypertension, he must have high blood pressure. And since the little we eat is the little we get from the people that help us, then you can imagine, what else can we do (…) we are both taking [the medication] to at least protect us a little from something. − Female, 55, immigrant, street vendor, income below BMS (Quito)

While one’s position within the social hierarchy clearly mediates the ability to access healthcare and achieve continuity in pharmacological treatment, we must also consider the social and political mechanisms that generate, configure, and maintain such a social hierarchy (Figure 1). A significant proportion of the participants reported having no source of income during the pandemic and being unable to purchase medication, which can be viewed in relation to the lack of protective labour policies that regulate informal working arrangements, or the limited reach of anticipatory policies that provide social protection to people during health emergencies. Participants described the limited reach of government support.

The government gave a subsidy of 60 dollars for three months, here in my area, I think, you see, in this area, in this sector, only two people received it. − Male, 61, farmer, income below BMS (Esmeraldas)

Even those in formal employment mentioned periods of reduced salary under the ‘humanitarian law’ or even times without pay during the pandemic.

Yes, it has affected us financially, it has affected us as people, it continues to affect us because the state owes us all the teachers [our salary] for two months now, so financially it has affected us a lot. − Female, 43, teacher, income above BMS (Esmeraldas)

Furthermore, frequent stock-outs of essential medication in public health facilities must be considered a political issue related to poor planning, governance and/or supply chain issues. According to one participant who worked in health services, the stock shortages were not necessarily a new phenomenon caused by the pandemic but were exacerbated during that time.

… sometimes there is no medicine in the pharmacy, so to continue with the treatment we have to go out to find it, to buy with our own money. − Male, 58, lab assistant, income above BMS (Esmeraldas)

## Discussion

Our study highlights the difficulties with continuity of chronic disease care during the initial months of lockdown and serious issues with basic medicine supply in the public health system which have also been identified in other low-middle income countries.(Singh et al., 2021; Tullo et al., 2020) This failure in supply intensified the population’s reliance on out-of-pocket payments to cover their treatment costs, which, during a time with reduced or absent earnings due to interrupted employment, had a catastrophic impact on the most vulnerable populations. Poverty and poor health are highly intertwined, especially in settings with low-income levels, where there is a tendency for worst health outcomes (Wagstaff, 2002) and higher barriers to access medical treatment for chronic diseases like diabetes (Christiani et al., 2016). In line with this, individuals with informal employment and low educational status frequently reported going various days or months without medication. The issue emerged most poignantly with one individual describing the need to choose between purchasing food or purchasing medication.

The economic impacts of the pandemic in low- and middle-income countries must be considered within the overarching structural determinant which is the limited reach of social policies to support the population during health or other emergencies. In contrast with European and other high-income nations where furlough schemes provided workers with a source of income during the lockdown (Andor, 2021), more than 60% of workers Ecuador are in the informal sector, (Canelas, 2019) and a significant proportion of the population had no source of income during the pandemic (Jara et al., 2022). While material barriers to accessing healthcare included financial restraints and limited mobility due to reduced public transport schedules, participants also discussed their reticence to go to the health facilities due to fear of contagion, a factor that has been identified in other contexts (Arruda et al., 2018; Gutiérrez et al., 2016; Tolosana, 2018). Their heightened perceived vulnerability can be linked to widespread understanding that diabetes and hypertension are risk factors for a poor COVID-19 prognosis.

Our study revealed critical differences between the urban and rural areas in line with other studies (De Coninck et al., 2020; Rodríguez-González et al., 2020). Even though rural areas tend to present more material barriers to access healthcare due to the limited transport, long distances and lower availability of services, the role of lay health promoters made a positive difference by continuing care even during the pandemic and thus improving the management of the chronic disease. This advantage was lacking in Quito, where health promotion is more focused on activities organised in the health centre, such as dance therapy, which were completely cancelled during the pandemic. One participant in Quito identified a chat group organised by the Diabetic Patient’s Club at her primary health centre as a gateway for patients to consult and share concerns with their peers. The use of social media groups as a way to provide and receive psychosocial support may be crucial in an urban lockdown situation, where social support is limited (Kingod et al., 2017).

Social support was identified as a cornerstone of NCDs management during COVID-19 by our participants. When lacking economic resources, many participants depended directly on their families to continue with the pharmacological treatment. Our findings suggested that rural communities had a strong community support network relying frequently on family, neighbours or friends to help them to overcome system shortfalls. This contrasted with the thinner support network more frequently reported by participants from Quito, perhaps due to the anonymity that characterises urban centres. In this context, vulnerable people with impeded accessibility and unanswered calls were more likely to be isolated. Further research should be conducted to explore the role of family and community support to promote access to healthcare during the pandemic and beyond. Another remarkable finding was the increased use of traditional medicine during the pandemic, either as the sole remedy or combining it with the conventional medical treatment. Traditional medicine is widely used in Ecuador, above all in the rural context due to the culture and accessibility to plants (Kasole et al., 2019), but also as an alternative to healthcare under unfavourable conditions related to cost, availability or accessibility, such as during the COVID-19 pandemic (Gallegos Zurita, 2016).

This study has several limitations. Firstly, due to the recruitment methods, our sample included participants with some form of contact with the health system (patient clubs in Quito and lay health promotors in Esmeraldas) and access to a personal telephone. Given that the study is exploring issues with healthcare access, it is possible that we underestimate the true severity of the issue. The participants may experience fewer barriers to healthcare access than patients that do not attend patient clubs or people who live in other remote areas where there are no lay health workers in the community. That being said, the challenges experienced by the participants of this study are real and cause for concern in themselves. Furthermore, we experienced recruitment limitations in rural areas regarding the ability of the research team to contact community members, particularly in areas with limited mobile network reception. This has influenced the number and profile of rural participants reached in our study, with most of them having an income above the BMS probably due to their intermediate-level occupations. While they also endured difficulties during the pandemic due to the humanitarian law, they may have benefited from their stronger social support network to overcome problems in access to healthcare. Therefore, we can expect that people from rural areas with a lower socioeconomic situation faced higher difficulties not collected here. Despite the lower percentage of rural participants, all shared similar narratives that allowed data saturation. Secondly, the interview extracts were translated from the local language, Spanish, to English, and as such, it is possible that some nuances in the expressions used have been lost. To minimise this issue, we ensured that translations were reviewed and discussed with native speakers of both Ecuadorian Spanish and English. Lastly, conducting the interviews via telephone may have impacted the information provided by the participants, being more or less forthcoming about certain issues. Furthermore, it meant that we were unable to analyse their visual expressions and/or body language.

It is worth mentioning that the WHO’s framework of social determinants of health has been widely questioned within the critical epidemiology discourses of Latin America due to its causalist and deterministic nature. A counter-hegemonic discourse has been proposed based on the Theory of the Social Production of Health (Breilh, 2013; Rocha & David, 2015; Spiegel et al., 2015) which addresses the social determination of health as a theoretical framework incorporating the collective and the social-historical nature of the health/disease phenomena (Rocha & David, 2015). While recognising these criticisms, our choice of the WHO framework was based on the fact that it offers a pragmatic way of framing people’s lived narratives according to the multidirectional causes of health inequities and allows us to conceive the hierarchical social dynamics within the system. However, we recognise that the framework employed may leave out the dialectical analysis of social processes and the critique of systems of oppression that have led to the social injustices exacerbated by the COVID-19 pandemic in the first place.

Our study shows, from the patients’ perspective, how the COVID-19 response affected healthcare access for people with diabetes and hypertension in Ecuador. Healthcare reorganisation and a shortage of medication left patients with discontinued management of chronic diseases and increased out-of-pocket expenses. Financial restraints, limited mobility and fear of contagion were key barriers to continuing chronic disease care, while support from family members and use of traditional medicine were frequently reported as ways to overcome challenges. The lack of protective labour policies and limited reach of anticipatory policies for health emergencies likely worsened pre-existing health inequities. It is important for policymakers and public health professionals to address the presented impacts of the pandemic on healthcare access for chronic conditions and prevent widening pre-existing health inequalities.

## Supplementary Material

S1

S2

## Figures and Tables

**Table 1:** Characteristics of the participants.

	Quito, *n* (%)			Esmeraldas, *n* (%)	
Characteristics	Men	Women		Men	Women
**Age in years, median (range)**	61 ± 7	54 ± 5		58 ± 3	56 ± 16
**Condition**					
Type 2 Diabetes	6 (86.0)	5 (83.3)		1 (33.3)	2 (66.6)
Hypertension	0 (0.0)	0 (0.0)		1 (33.3)	1 (33.3)
Both	1 (14.0)	1 (16.7)		1 (33.3)	0 (0.0)
**Disease history**					
0–9 years	0 (0.0)	2 (33.3)		1 (33.3)	1 (33.3)
10–19 years	3 (43.0)	2 (33.3)		1 (33.3)	2 (66.6)
20 years or more	3 (43.0)	0 (0.0)		1 (33.3)	0 (0.0)
Not reported	1 (14.0)	2 (33.3)		0 (0.0)	0 (0.0)
**Education**					
Primary	3 (43.0)	2 (33.3)		0 (0.0)	1 (33.3)
Secondary	3 (43.0)	3 (50.0)		3 (100.0)	0 (0.0)
Tertiary	1 (14.0)	1 (16.7)		0 (0.0)	2 (66.6)
**Occupation**					
Technicians and professionals at intermediate level (e.g. teachers)	0 (0.0)	0 (0.0)		1 (33.3)	2 (66.6)
Elementary occupation (e.g. cleaners, unpaid housework)	0 (0.0)	1 (16.7)		0 (0.0)	1 (33.3)
Construction and crafts	3 (43.0)	0 (0.0)		1 (33.3)	0 (0.0)
Services and sales (e.g. taxi drivers, street vendors)	2 (29.0)	4 (66.6)		0 (0.0)	0 (0.0)
Agriculture	1 (14.0)	0 (0.0)		1 (33.3)	0 (0.0)
Unemployed	1 (14.0)	1 (16.7)		0 (0.0)	0 (0.0)
**Monthly income**					
Below $400	6 (84.6)	5 (83.3)		1 (33.3)	1 (33.3)
Above $400	1 (14.0)	0 (0.0)		2 (66.6)	2 (66.6)
Not reported	0 (0.0)	1 (16.7)		0 (0.0)	0 (0.0)
Total	7 (100)	6 (100)		3 (100)	3 (100)
