# Supplementary material for: Access to health services for chronic disease care during the COVID-19 pandemic in Ecuador: A qualitative analysis using a Social Determinants of Health approach
Source: Glob Public Health. Author manuscript; Available in PMC 2024 Sep 23. (PMC7616628; doi:10.1080/17441692.2023.2224859)
Supplement: S1 [file EMS198626-supplement-S1.pdf]

La determinación social de la salud como herramienta de transformación hacia una nueva salud pública (salud colectiva)

The social health determination as a tool of transformation towards a new public health (community health)

Jaime Breilh[1]

[1] Md. MSc. PhD, Director del Área de Ciencias de la Salud de la Universidad Andina Simón Bolívar, Coordinador del Doctorado en Salud Colectiva, Ambiente y Sociedad. Correo electrónico: jaime.breilh@uasb.edu.ec

Recibido: 02 de febrero de 2013. Aprobado: 04 de abril de 2013.

Breilh J. La determinación social de la salud como herramienta de transformación hacia una nueva salud pública (salud colectiva). Rev. Fac. Nac. Salud Pública 2013; 31(supl 1): S13-S27.

Posicionamiento epistemológico de partida y breve recorrido histórico de la propuesta

La *determinación social de la salud* es una de las tres categorías centrales de la propuesta para una epidemiología crítica, enunciada desde mediados de la década del 70 y luego desarrollada y complementada por otros autores especialmente de la corriente latinoamericana.Junto con las categorías *reproducción social* y *metabolismo sociedad-naturaleza* han conformado el eje teórico de una propuesta de ruptura con el paradigma dominante de la salud pública.

El punto de partida de esta construcción teórica se fundamenta en una relectura -desde el realismo crítico- de dos vertientes epistemológicas encabezadas por Kuhn y Bourdieu. Del primeropara rescatarla visión dialéctica del pensamiento científico, su naturaleza social, transformabilidad y el carácter "revolucionario" dado por la oposición de ideas innovadoras frente a los aspectos "normales", lo que abre puertas a una aproximación sociopolítica de la determinación comunitaria de la ciencia, sus modelos e instrumentos [1]. Y el segundo, porque analiza "…la teoría como un modus operandi que orienta y organiza la práctica científica...(y las)…capacidades creadoras, activas, inventivas del *habitus...*" y el lado activo del conocimiento científico como "…el capital de un sujeto trascendente...de un agente en acción" [2].

Con esto se plantea que el conocimiento epidemiológico, más allá de sus proyecciones técnicas, y como toda otra disciplina científica, hace parte del poder simbólico, que es el poder de constituir, "…de enunciar, de hacer ver y hacer creer, de confirmar o de transformar la visión del mundo, y de este modo la acción sobre el mundo…un poder casi mágico que permite obtener el equivalente de aquello que es obtenido por la fuerza" [2]. En efecto, un poder tan importante, que ha logrado hasta cambiar el curso de la historia como sucedió con la campaña de fiebre amarilla de la Fundación Rockefeller

* Este artículo se basa en apartados de la conferencia presentada en el "VIII Seminario Internacional de Salud Pública: Saberes en Epidemiología en el Contexto del Siglo XXI" realizado en la Universidad Nacional, Bogotá, marzo de 2013.

y su impacto para el giro de la Revolución Mexicana frente a la presencia de la petrolera Standard Oil [3].

Por eso la epidemiología es un terreno de lucha de ideas, de disputa sobre cómo enunciar la salud y cómo actuar, y esa disputa obedece a intereses sociales encontrados. En esa medida la disciplina comparte con todas las demás, la característica consustancial de toda ciencia de ser una operación simbólica, y por lo tanto es "… una expresión transformada, subordinada, transfigurada, y algunas veces irreconocible de las relaciones de poder de una sociedad" [2], y en nuestro caso, dichas relaciones implican la imposición de un sistema de explotación social y de la naturaleza, que reproduce sociedades no sustentables, inequitativas y malsanas.

Al asumir una tarea de reformulación disciplinar es inevitable situarse en una perspectiva teórico-política, es decir, mirar desde un ángulo específico de dichas relaciones de poder, o desde lo que suele denominarse un punto de vista social, de clase; perspectiva que imprime una huella profunda en la construcción del conocimiento, aun de sus expresiones más técnicas.

Reconocer aquello, saber que cuando se hace epidemiología hay una politicidad implícita y un nexo evidente o tácito con intereses estratégicos de un sector social, entonces, no solo es un acto de transparencia ética, sino un signo de rigurosidad metodológica. Cuestión básica, sobre todo cuando el objeto de estudio, constituye un elemento sensible de la evaluación de un proyecto social o de gobierno, por ser un recurso "diagnóstico" con el que se define la imagen sanitaria de la sociedad, con el que valoramos el éxito o fracaso de las políticas y, sobretodo, con el que construimos evidencias de los impactos generados por la producción y el vivir social.

En trabajos previos a través de una amplia revisión documental ha sido posible señalar los afanes de grandes empresas y el papel de una epidemiología bajo contrato, aplicada para fabricar la duda del público y de la justicia, sobre los impactos humanos y ambientales de producciones peligrosas, mediante manipulación estadística y otras formas de dilución de evidencias; maniobras para implantar la idea de "otros factores causales"; y operaciones técnicas para crear incertidumbre por supuestas fallas en diseño o de datos [4].

Si bien los casos descritos son ejemplos extremos de una epidemiología venal, la mayor parte de veces las operaciones de una epidemiología funcional no se trata de nexos tan directos, sino que la epidemiología convencional, incluso con las mejores intenciones, estructura su discurso metódico de manera que hace invisibles las relaciones de determinación generadas por el sistema económico de acumulación de capital, las relaciones de inequidad que lo reproducen y la destrucción de la naturaleza.

Es decir, el análisis de los paradigmas y modelos con los que ha trabajado la epidemiología, se encuentra inevitablemente atravesado por las tensiones, impulsos y obstáculos que resultan de esas relaciones de la ciencia con el poder, de lo cual ha resultado una secuencia de confrontaciones en cada período histórico. Confrontaciones que no se subsanan con la adición de un reconocimiento de "causas de las causas" y de los "determinantes estructurales", mientras sigue inserta en la misma lógica empírico-funcional.

El pensamiento crítico latinoamericano en la mayoría de casos bebió de dos fuentes importantes del pensamiento crítico epidemiológico: las ideas revolucionarias sobre determinación de la salud de personajes que inspiraron la lucha anti-colonial en el Siglo XVIII [5]; y las obras revolucionarias de Europa del Siglo XIX que explica Howard Waitzkin en su crítica sobre "la medicina y la salud pública al final del imperio" [6]. En Norteamérica el hilo conductor de un razonamiento epidemiológico crítico del sistema social e integral se plasmó en obras como las de Henry Sigerist [7], o un poco después en las de George Rosen [8].

Bajo el influjo social de los 70 se crearon las condiciones para que varios núcleos de la medicina social de América Latina se enfocarán en la relación entre el orden social capitalista y la salud, escenario en que nos fue posible proponer de manera directa y pormenorizada el uso de la noción de *determinación social* de la salud en la epidemiología. Categoría que desarrolla una crítica del paradigma empírico-funcionalista de la epidemiología y propone una herramienta para trabajar la relación entre la reproducción social, los modos de vivir y de enfermar y morir [9].

Herramienta teórico metodológica que alimentó y animó la construcción de la epidemiología crítica latinoamericana, forjada en el debate y la ruptura con el canon de la epidemiología clásica y su multicausalismo lineal, luego frente al modelo empírico-funcionalista de la epidemiología ecológica, y ahora, frente al de la epidemiología de los llamados "determinantes sociales de la salud".

Cabe aclarar que la *epidemiología crítica latinoamericana* si bien se fraguó en escenarios académicos, tuvo siempre como fuelle y motivo de inspiración la lucha de nuestros pueblos por superar un régimen social centrado en la acumulación de riqueza, y no las preocupaciones burocráticas de la llamada gobernanza.

El carácter crítico de la propuesta se fundamenta en la vinculación con las luchas de los pueblos por superar un régimen social centrado en la acumulación de la riqueza y el reconocimiento de la incompatibilidad del sistema social imperante y el modo de civilización que lo reproduce, frente a la construcción de modos de vivir saludables. No es su objeto descifrar conexiones empíricas para promover medidas de simple reforma y representar la entelequia de la salud en una sociedad estructuralmente patógena. La epidemiología crítica no puede ser otra cosa que radicalmente emancipadora.

Finalmente y por todo lo que queda dicho, la noción de *determinación social de la salud,* como toda idea de ruptura, no es el producto aislado de elucubraciones

personales sino la expresión del pensamiento crítico que se había apoderado del movimiento latinoamericano de medicina social (ahora mejor conocido como de salud colectiva); a cuyo origen convergieron las inquietudes de colectivos movilizados, el desarrollo de nuevos instrumentos de análisis y la presencia de un proyecto expreso de transformación del paradigma obsoleto de la vieja salud pública empírico-funcionalista de los años 70.

Entre los años 70 a 90 desde el nuevo pensamiento epidemiológico latinoamericano se realizaron cuestionamientos a la epidemiología lineal [10-20], lo que contribuyó a refundar la moderna epidemiología crítica, y que ha sido ampliamente comentada [21-24] y documentada [25]. El aporte inicial se orientó a descifrar como elempirismo cognitivo, la metodología positivista y la lógica cartesiana de la epidemiología causal, constituyeun obstáculo para penetrar la génesis de la salud [9], y como la noción de causalidad de Humese impuso como lógica rectora del pensamiento epidemiológico en la corriente hegemónica de los 70 [26] y los desarrollos más recientes [27].

La perspectiva *cartesiana* había penetrado en esos enfoques del análisis epidemiológico, reduciendo la realidad al sólo plano de los fenómenos; asumiendo las propiedades de las partes (individuos) como base del comportamiento del todo; y otorgando, de ese modo, prioridad interpretativa a los atributos individuales (la parte) sobre el todo [28].

A partir de ese marco, la epidemiología convencional cayó en una distorsión múltiple de la metodología, posible de sintetizar en los siguientes cinco elementos:

- Una explicación fenoménica, reduccionista y fragmentaria de la realidad y la determinación de la salud.
- Primacía absoluta de la asociación causa-efecto como gran organizadora y lógica del universo epidemiológico.
- Reificación de la relación causa-efecto como artefacto formal aplicado para identificar factores de riesgo.
- La reducción de la noción de exposición/vulnerabilidad a un problema esencialmente individual de naturaleza probabilística.
- A la hora de organizar la práctica epidemiológica, la reducción de ésta en acciones funcionales sobre factores de riesgo.

A esta epidemiología empírica, a pesar de su robusto arsenal formal estadístico, no le es posible explicar la relación entre el sistema social, los modos de vivir y la salud; ni entender la distribución por clases de las formas e intensidades de exposición humana a procesos peligrosos; ni la vulnerabilidad diferencial de los colectivos situados en inserciones sociales distintas; ni comprender en profundidad el metabolismo sociedad-naturaleza y su impacto social. Y finalmente, con este modelo, resulta imposible "conocer la sociedad para transformarla" hacia una salud profunda, que fuese el pilar no sólo de una nueva salud pública, sino de la construcción de un nuevo modelo civilizatorio saludable.

Las posibilidades de un análisis crítico de la salud se diluían en las limitantes construcciones empíricas de *lugar, persona*, y *tiempo*. Y al buscar una apertura hacia las relaciones de la salud con el contexto de la vida, se proponía la solución de la epidemiología ecológico-empírica, basada en los principios parsonianos de la teoría de los sistemas en equilibrio[†] que congelaba la relación entre *agente, huésped* y *ambiente* en un proceso de *historia natural de la enfermedad* [29]. Como lo señaló acertadamente Donnangelo dicha teoría sirvió como instrumento para tejer el camino de lo social (i.e externo) a lo comportamental-individual, consolidando de ese modo la lógica funcionalista [30].

Con este tipo de herramientas no es comprensible un orden social que mercantiliza la naturaleza, ni el rico movimiento dialéctico que se da entre lo natural-biológico y lo social, pues el molde estático y rígido de la ecología empírico-funcional reproduce la cosmovisión positivista, que separa al sujeto de un mundo de objetos que lo rodean, y convierte las relaciones *sociedad-naturaleza* y *social-biológico* en meras externalidades entre factores, variables de la realidad, creando una falsa relación substitutiva de conexión externa de variables. *Lugar* y *ambiente* aparecían, desde esa perspectiva, como categorías que representaban un mundo atomizado, de fenómenos empíricamente evidenciables, donde la relación social-natural era una más de las conexiones formales que había que incorporar al modelo epidemiológico estadístico.

Como respuesta a esta perspectiva se produjeron trabajos críticos amparados en el ambiente intelectual propiciado en la naciente maestría en medicina social de la Universidad Autónoma Metropolitana – Xochimilco. Allí se ubica la tesis pionera que basó su análisis en la categoría *determinación social*, como una forma de superar el causalismo, en cuanto se refiere a la *producción o génesis* de la salud; la noción de *reproducción social* (en las dimensiones general-particular-singular) como superación del empirismo aplanado; la concepción dialéctica de la relación*social-natural-biológico*, como superación del ecologismo empírico; y que sometió a escrutinio crítico el uso empirista de las técnicas y la preeminencia de un enfoque cuantitativista del método [9].

[†] Talcott Parsons, teórico del estructural funcionalismo, explicó el mundo como un conjunto de sistemas y subsistemas con tendencia al equilibrio, y funciones que regulan los estados normales. El funcionalismo es una doctrina social que se ha pensado como antípoda de las ciencias de la transformación, sostiene la estabilidad eterna de la sociedad como un gran sistema con tendencia al equilibrio, mientras que el cambio sería como una pérdida momentánea de dicho equilibrio, que finalmente se reajusta por la operación de funciones que cada grupo social desempeña.

En cuanto a la *distribución* poblacional de las condiciones de salud, se propuso una operacionalización epidemiológica de la clase social, como superación de los estratos convencionales de la vieja epidemiología; para enlazar *producción y distribución* de la salud y exponer su complejidad, se propuso la categoría *perfil epidemiológico*, que sintetiza los dos ejes de dicha complejidad: el dimensionamiento general, particular y singular, y la antítesis entre procesos saludables, protectores o perfeccionantes, versus procesos malsanos, peligrosos, deteriorantes o vulnerabilizadores de la salud [9].

Este esfuerzo inicial fue enriquecido en los 80s con aportes como la matriz de triple inequidad en la determinación de la salud (clase, género y etnia); luego en los 90s con una recuperación del *sujeto* de la epidemiología, a través de la construcción intercultural de una crítica de la sociedad de mercado, y con la vuelta del siglo la investigación se enfocó al *metabolismo sociedad-naturaleza,* esbozado ya desde los aportes de los 70 [9], así como en la crítica de la inviabilidad del modo de civilización del capital para la salud y la naturaleza.

Con este recorrido se arriba al desarrollo más reciente, al planteamiento de las 4"s" de la vida–como se explica más adelante-, para confrontar la expansión violenta de la acumulación de capital, basada en los mecanismos devastadores de convergencia malsana de tecnologías; el despojo fraudulento de los recursos vitales de las sociedades subordinadas (i. e tierra, agua, genoma); y finalmente para situar en el contexto de esa matriz social devastadora los impactos de la triple inequidad inherente a las relaciones sociales y modos de vivir, que han sufrido los asalariados, subasalariados, los pequeños productores y aun la clase media [31-34].

El proceso ha implicado una permanente conexión entre el trabajo epidemiológico con la lucha para confrontar al poder patógeno del sistema social usando los instrumentos de la ciencia. Un esfuerzo por acompañar desde el quehacer académico la disputa de los sujetos políticos contra los peligros para la vida producidos por el capitalismo industrial y la marginalización naciente en los 70; posteriormente frente al agresivo posindustrialismo y desmantelamiento neoliberal de los derechos, que agravó la inequidad y exclusión social en los 80; y ahora en el siglo XXI contra la aceleración de la acumulación económica por convergencia de usos peligrosos de la tecnología productiva, despojo y shock, que ha modificado el perfil de sufrimiento humano y ha diversificado las formas de exposición masiva a procesos dañinos, amplificando y diversificando los mecanismos de destrucción de los ecosistemas.

Procesos que lamentablemente no están logrando realmente revertir ni los regímenes de América Latina que definen una línea progresista, pues empujan desde una falsa opción del extractivismo, sus afanes de una política social redistributiva que se busca forjar contracorriente al ritmo vertiginoso de concentración de riqueza y exclusión social de una maquinaria de acumulación de capital hasta ahora intocada. Por debajo de una intencionalidad social siguen reproduciéndose modos de producir y políticas que no afectan las raíces de una civilización individualista, que se sustenta en un complejo de irracionalidades (productivas, energéticas, ambientales), y que se reproduce y recicla gracias al imperio de un consumismo extremo, de un ordenamiento social dependiente de una matriz de derroche energético, de desperdicio de recursos vitales, y de una masiva e irreparable generación de desechos.

En todo este camino, una contradicción compleja ha sido el comprender cómo acoplar el debido reclamo de nuestro pueblo por acceso a los servicios asistenciales de la salud individual, con la urgencia –esa sí mucho menos comprendida e interiorizada-, de transformar con sentido preventivo la determinación social de los problemas de salud.

Rupturas para la construcción del método de la epidemiología crítica: lectura en paralelo con otras perspectivas epidemiológicas

La salud es un objeto polisémico en tanto objeto real,objeto pensado (de modo intersubjetivo) y campo de praxis. Avanzar hacia un paradigma crítico de la epidemiología, implica entrelazar tres transformaciones complementarias e interdependientes, el replanteo ontológico como *objeto* complejo, la innovación en su conceptualización en cuanto a las categorías y operaciones metodológicas que permitan hacerlo inteligible, y la apertura y vinculación con las formas de incidencia y las fuerzas sociales movilizadas [25], todo ello inscrito en un proceso dialéctico de determinación socio histórica.

Dada la conexión e interdependencia de objeto-sujeto-campo, la categoría determinación social (DS) aplica a los tres: hay una DS del objeto; una DS de la construcción de los conceptos y una DS de la práctica. En cada período histórico los cambios y movimientos de la salud como objeto, se interrelacionan con las innovaciones conceptuales y las transformaciones de la práctica (figura 1).

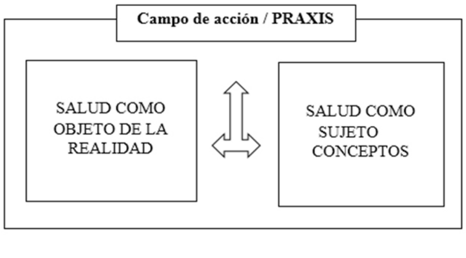

Figura 1. La salud noción polisémica

Dicho de otro modo, la transformación objetiva, subjetiva y práctica se producen socio históricamente en relación con una matriz social determinante; no hay manera de comprenderlas separadamente, como lo exponen algunos trabajos realizado en el Ecuador en los períodos de la Revolución anticolonial del Siglo XVIII [35], la Revolución social juliana de comienzos del Siglo XX (1925-1931) [36] y la etapa de regresión neoliberal agresiva de 1985 a 2006 [37], que permiten observar articulaciones muy diferentes en cada caso.

El conocimiento científico en cualquier disciplina se da en el movimiento entre sujeto y objeto en un campo de praxis; ninguna de los tres elementos define el método de manera absoluta. Entonces bien, al enfocar el objeto debemos apreciar que éste no existe como un "en sí" puro sin sujeto, ni tampoco existe un sujeto "en sí" puro sin objeto. El movimiento del conocimiento surge en la relación de los dos y esa relación se encuentra determinada por el movimiento práctico y momento histórico en el que se realiza.

Ahora bien, la relación entre sujeto y objeto requiere de la mediación de ciertas estructuras simbólicas, pues las experiencias perceptivas no están únicamente determinadas por las imágenes y estímulos de la naturaleza sino que dependen de la cultura, de esas estructuras simbólicas que aplicamos al conocer; una las cuáles es el método [38].

De ahí se desprende que en la epidemiología como en toda ciencia, el método es un movimiento que va del objeto al sujeto y viceversa, pero atravesado también por las ideas y la cultura. El método es una de las mediaciones simbólicas en el caso del conocimiento científico, y no es ni un movimiento exclusivamente inductivo reflejo (de objeto a sujeto), ni tampoco un movimiento exclusivamente deductivo (de sujeto a objeto). El método es un movimiento dialéctico.

Comprendido lo anterior, es posible explicar por qué el método empírico analítico (positivista) que aplica la epidemiología hegemónica es distorsionante, pues al acercarse a la realidad por la mera senso-percepción (procedimiento empírico inductivo de observar fenómenos mensurables –variables- y juntarlos bajo un sistema formal, matemático o no) sólo alcanza a mirar unas partes o fragmentos de la realidad (el pico del iceberg), tal y como ellas se registran en el acto de observar y medir efectos en el plano de los fenómenos -efectos epidemiológicos observables-, sin penetrar en el plano de la determinación de los procesos en el cual tienen lugar y adquieren sus propiedades dichos fenómenos.

Es por ello que se requiere un paradigma integrador, una operación para la que es indispensable un marco interpretativo del sujeto (categorías de un marco teórico) que, si bien son ideas, constituyen a su vez una expresión del mundo material y del trabajo de articulación lógica que posibilita la observación de dicha base material y la praxis.

Redimensionar el objeto de la epidemiología: ruptura con el empirismo lineal

Para discutir en torno el objeto de la epidemiología, como objeto real y sus propiedades, se utilizan algunas categorías que permitan caracterizar y comparar la forma como esto es definido en los más representativos paradigmas epidemiológicos [39][‡], como son: el *lineal causal –clásico-* (epidemiología lineal uni y multi causal); el ecológico-empíricos (teoría parsoniana de los sistemas agente, huésped y ambiente); el causal de *transición* (epidemiología social de los determinantes sociales); y cuatro expresiones de la epidemiología crítica (medicina social, epidemiología etno-social, epidemiología eco-socialy epidemiología crítica). Las cuatro categorías analíticas son:

- Orden. Concepción acerca de qué es lo que mueve al objeto, es decir el orden social y sus relaciones de jerarquía.
- Carácter del movimiento. Visión de cómo se da su movimiento, génesis y reproducción.
- Temporalidad. Concepción de la temporalidad del objeto, sus elementos e historicidad.
- Espacio social y elementos. El espacio social de la determinación y la espacialidad del objeto (dimensiones, conexión histórica de elementos).

En cuanto al *orden* la conceptualización de la DS pasa por la idea de orden social –qué es lo que mueve el objeto. En la epidemiología *lineal clásica (uni o multicausal)* el orden es causal-individual y la jerarquía determinante se expresa en los principios de conjunción/asociación de Hume –que luego Bradford Hill sistematizó [40]. Desde esta perspectiva la operación eficiente de una causa gracias a las conexiones externas de ésta con el efecto es lo que incide en efectos fenoménicos individuales; el principio del movimiento es la causación por conjunción o conexión externa con factores de un ambiente cosificado y fragmentado, una concepción del orden que conlleva a concluir que la acción de la salud pública es igualmente causal sobre factores de riesgo (figura 2).

No importa cuanto se sofistique el sistema causal sigue siendo lineal y reduccionista, pues ni la formación de una red de causas -como en el modelo de MacMahon puede expresar la complejidad y el movimiento; y más

[‡] Para los fines de este análisis se han escogido aquellos textos que corresponden en su inicio a los años 70, que podrían asumirse como iniciadores del paradigma, sin que en este preliminar inventario epistemológico incluya todos los que deberían estar.

aún, la cosificación de los procesos en factores se brinda para convertir realidades determinantes como la clase social en elementos cosificados y marginales de dicha estructura social (figura 3) [26].

Por otra parte la epidemiología *empírico ecológica de sistemas* (tríada ecológica de Leavell & Clark) asume como orden el equilibrio de sistemas y subsistemas conectados, y la jerarquía está dada por las pulsiones de equilibrio y función [29] (figura 4).

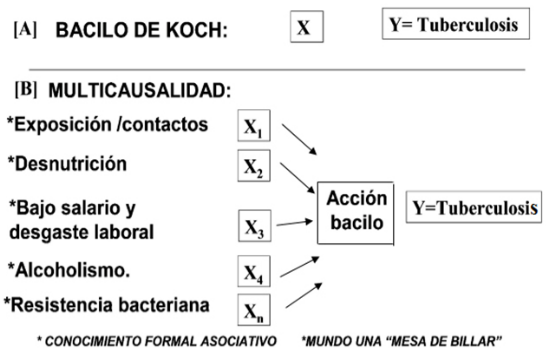

Figura 2. Modelo lineal causalista de la tuberculosis

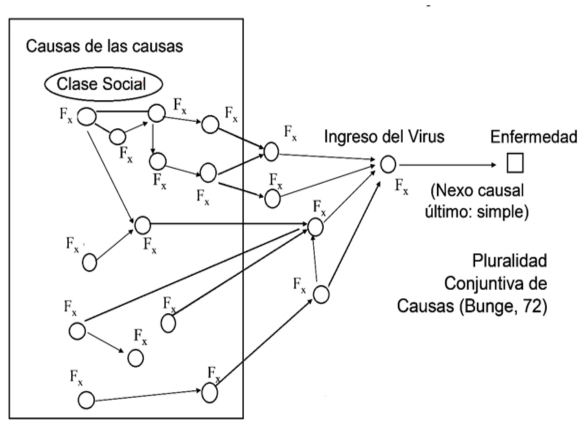

Figura 3. Modelo lineal multicausalde la hepatitis
Fuente: Breilh J. Epidemiología: Economía Política y Salud, 1979.

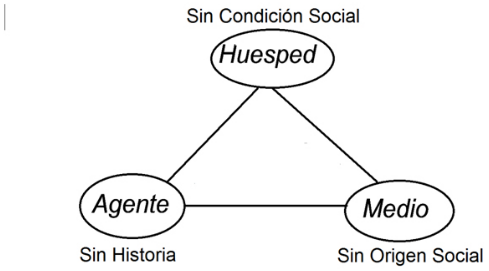

Figura 4. Epidemiologia empírico ecológica de sistemas
Fuente: Historia natural de la enfermedad Leavell y Clark, 1965.

Pasando luego a los modelos de la epidemiología causal de transición –aquí la noción de transición se refiere a la apertura a asumir las estructuras sociales como parte del objeto. La obra de Mervin Susser es un paso inicial de esta línea. Reorganiza el causalismo en el marco de la teoría de sistemas, planteado de este modo:

> "La evolución de los modelos causales en epidemiología nos ha llevado a la noción de sistema….los sistemas se relacionan mutuamente, contienen el uno al otro…. El universo tiene una existencia simultánea y cada nivel de organización está incluido en otro más complejo. Los átomos están contenidos por las moléculas, las moléculas por los cromosomas, los cromosomas por las células, y las células por los tejidos. Los órganos y sistemas fisiológicos están contenidos por los individuos y los individuos en los grupos sociales…Todos estos sistemas se hallan ligados [41].

A la luz de este razonamiento sistémico, la acción "depende de si tenemos en mente el miasma o los microorganismos, enzimas, moléculas, conducta humana o la estructura de las sociedades" con lo cual se expresa ya en 1973 una lógica que se anticipa a la de los impulsadores del paradigma de los determinantes sociales en el 2007, los determinantes estructurales como una estructura causal externa [41].

De hecho, el paradigma de la llamada *epidemiología social* propugnado por la Comisión Mundial de la OMS sobre "determinantes sociales de la salud" es otro causalismo que explica también el orden bajo la noción causal. Representa un paradigma de transición crítica por que se abre a lo estructural, rompiendo el cerco de la visión más restrictiva de la epidemiología convencional, sin que represente un cambio de paradigma, en tanto convierte las estructuras sociales en variables y no en categorías de análisis del movimiento histórico de la acumulación.

Con esto no es posible ofrecer una crítica directa de la organización social de la sociedad de mercado y de sus rotundas consecuencias en la salud; ni analizar el proceso radical de acumulación económica/exclusión social, como eje de una reproducción ampliada de la inequidad social, ni abordar el metabolismo sociedad naturaleza (figura 5). Escogen la vía causal deificando categorías analíticas en factores o *causas estructurales*, que aparecen como abstracciones sonoras pero vaciadas de contenido crítico y de movimiento: *determinantes estructurales de inequidades* (gobernanza, políticas, cultura) y unos *determinantes intermedios* (circunstancia materiales, conductas y factores biológicos) [42].

Los organizadores teóricos de este modelo [42-44], asumen las dimensiones de lo general, particular y singular, como se propuso en América Latina, pero convirtiéndolas en niveles de variables o factores causales, armandoun modelo para trabajar limitadas y funcionales acciones de gobernanza, si bien proyectan una crítica so-

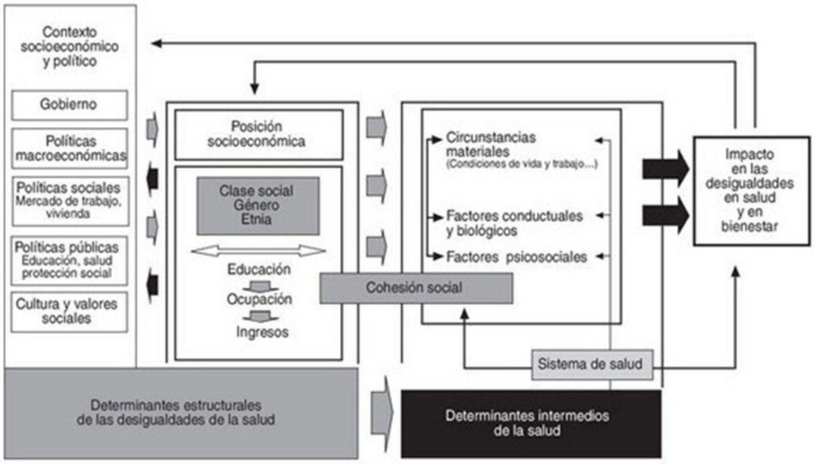

Figura 5. Modelo de determinantes sociales de la salud de la OMS.
Fuente: Solar e Irwin, 2007.

cial y el discurso de una epidemiología renovada, recaen en los vicios interpretativos y limitaciones del causalismo.

En términos metodológicos presentan sus explicaciones más como un esquema formal de articulación de *variables*, y no se logra un modelo teórico categorial-capaz de interpretar el sistema social de mercado,en su estructura de propiedad, sus procesos de generación y reproducción de la explotación humana y de la naturaleza, ni logran identificar las correspondientes jerarquías de subsunción [45] entre el sistema social, los modos de vivir de los grupos estructuralmente constituidos y los estilos de vida de las personas con sus familias.

En la producción de dicho modelo se encuentran *ideas fuerza* de la producción crítica latinoamericana que circularon tempranamente por los canales de la salud pública progresista de la región e incluso por algunos del *mainstream* epidemiológico, aunque sus autores no reconocen las originales contribuciones latinoamericanas. La propia categoría determinación social, terminó de ese modo transmutada en *causa de las causas*; es decir, la noción emancipadora de *determinación social* terminó convertida en la noción funcional de *determinantes sociales*, aptos para modificaciones de la gobernanza.

En el terreno de los paradigmasde la epidemiología crítica encontramos cuatro paradigmas. Dos de éstos, el de Laurell y el nuestro, se posicionan en el realismo crítico materialista del siglo XIX, aunque ofreciendo sustanciales innovaciones. Los otros dos, la etno-epidemiología de Almeida Filho y la eco-epidemiología de Krieger, retoman algunos elementos de los dos anteriores y se posicionan desde el pensamiento Habermasiano, y especificándose a una etnografía critica en el primer caso, y en una ecología política con tesis emancipadoras de género, en el otro.

La perspectiva de la epidemiología eco-social [46-48] recupera la importancia de trabajar la salud en sus múltiples dimensiones y hace su propia lectura de categorías como *subsunción*y de la noción de *metabolismo de sociedad y naturaleza* y plantea la noción de *embodiment* (*encarnación*) y lo que llama las rutas de *embodiment*, que las explica como caminos en que se da la encarnación de las exposiciones y vulnerabilidades socialmente generadas en los organismos [49] (figura 6).

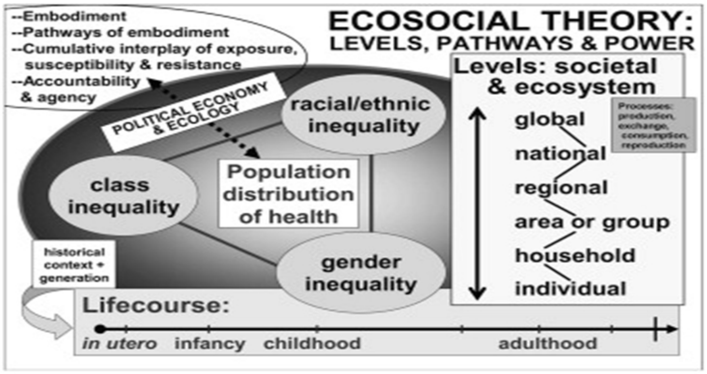

Figura 6. Modelo Eco-social y encarnación de inequidad Krieger
Fuente: Krieger, N. Epidemiology and the people's health: theory and context, 2012.

En la dimensión práxica de la propuesta de Krieger el eje lo ocupan las nociones de rendición de cuentas o responsabilidad (*accountability*) y agencia o acción (*agency*) ligada a las disparidades e inequidad. A pesar de su valor, el desafío supera las posibilidades de estas nociones pues se trata de la transformación de las relaciones de poder construidas alrededor de un sistema acumulación y exclusión, que destruye la vida, y por tanto algo más complejo, que amerita el enfrentamiento estratégico, sobre una base popular, para una superación del sistema de contradicciones, y que en la propuesta de Breilh se organiza bajo la categoría perfil epidemiológico.

La diferencia con esta noción, en la epidemiología crítica de Laurell y Breilh, es un alejamiento de la noción lineal de ruta (*pathway)*, planteando una comprensión dialéctica de proceso de determinación por generación y reproducción, donde opera el movimiento entre subsunción y autonomía relativa, para soslayar una posible interpretación lineal del movimiento de determinación (figura 7).

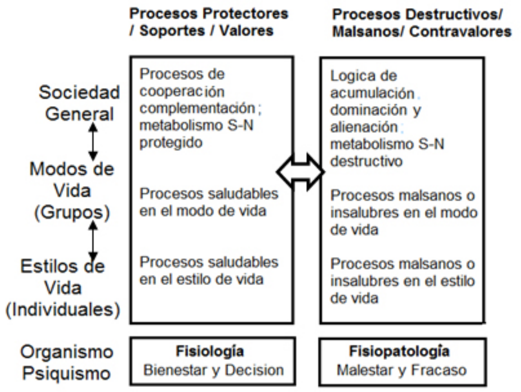

Figura 7. Perfil epidemiológico
Fuente: Breilh, 1976/1977

Como diría Donnangelo, desde este paradigma se incorporan las nociones de proceso y de lo colectivo. Mientras en la epidemiología convencional se enfoca fundamentalmente procesos individuales y lo social aparece apenas como "variables" externas que inciden en estados patológicos de personas; la epidemiología social asume los procesos colectivos o grupales como elemento irreductible a lo individual, y espacio de intervenciones [30].

De ahí la necesidad de asumir una lógica dialéctica que mantenga con vida los dos polos del movimiento de determinación (individual y colectivo) pero entendiendo el papel de cada uno. Un movimiento que Samaja describió acertadamente y que deja con vida los dos polos del orden: un proceso de generación de cambio que va de lo micro (individuo, más simple) hacia lo macro (social, más complejo); al que se opone dialécticamente un proceso de reproducción de las condiciones generales de la sociedad.

Visto así el orden de cosas, la salud no obedece a un orden exclusivamente individual, sino un que es proceso complejo, socialmente determinado, aspecto que muchas veces desaparece del pensamiento en la salud pública, debido al predominio de una visión biomédica, que reduce la problemática al estrecho límite de los trastornos o malestares individuales, su curación y la prevención individual.

La determinación social va y viene dialécticamente entre las dimensiones general, particular y singular: se reproduce de lo general a lo particular, y se genera de lo particular a lo general.

En el proceso se dan formas de subsunción en las cuales los procesos de la dimensión más simple se desarrollan bajo subsunción respecto a las más complejas y entonces aparece que la relación social-natural, o social-biológico ocurre como un movimiento entre partes de un todo concatenado que es la naturaleza (figura 8). Claro que las partes implican niveles de complejidad variables, sujetos a ciertas condiciones dinámicas que tienen una estabilidad apenas relativa.

En el movimiento de la naturaleza y sus sistemas, la tendencia central no es la de un proceso de adaptación y tendencia al equilibrio, tampoco es una relación externa entre diferentes sistemas, lo que hay es un movimiento de cambio permanente del patrón de transformaciones mutuas que se establece entre los organismos y el ambiente [28].

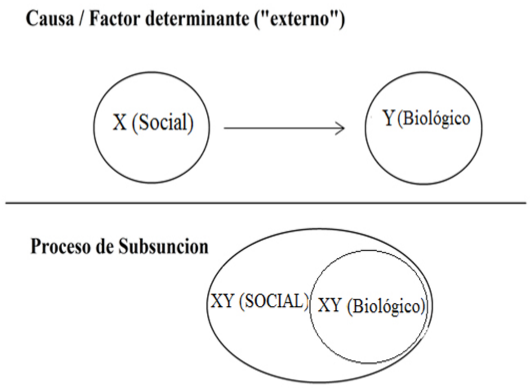

Figura 8. Subsunción: superación de la concepción lineal sobre la relación biológico- social
Fuente: Breilh, 1977/94

El redimensionamiento del sujeto de la epidemiología

La interdependencia entre los tres elementos del conocimiento vuelve a mostrarse al analizar el desarrollo de las concepciones del sujeto en la epidemiología, un movimiento que implica diferencias notables

§ Habitus: categoría recuperada para la epistemología por Bourdieu (El poder simbólico, 1998) para "tratar la teoría como un *modus operandi* que orienta y organiza prácticamente la práctica científica.

no sólo en las nociones de identidad del sujeto y en sus concepciones generales sobre la realidad y la metodología, incluso en cambios radicales en las concepciones uniculturales y antropocéntricas que impregnaron el pensamiento académico.

Al contrastar los modelos epidemiológicos analizados, se pueden establecer contrapuntos en la forma de asumir cada uno de los atributos que dan cuenta de la concepción acerca del sujeto, para lo cual se proponen las siguientes categorías:

- Identidad. Rasgos propios frente a la colectividad y sociedad; conciencia de clase, de género y etnocultural; conciencia de unidad y diversidad
- Concepción y relación con la naturaleza. Concepción sobre relación sujeto-naturaleza, separación o unidad S-N; visión de la importancia de la relación S-N para la salud y la vida; conciencia antropo, bio o sociobio céntrica.
- Recorte metodológico. De campo problemático, planos de la realidad y relación cuali-cuantitativo.
- Elemento activo del conocimiento.Preeminencia y centralidad de objeto, sujeto, praxis o su interdependencia.
- Criterio de verdad. *Habitus*[§] metodológico que permite demostrar las afirmaciones epidemiológicas veraces.
- Posición ética. Ethos de la práctica, en el sentido de forma característica e inclinación de “hacer el bien”
- Paradigma general de sustento. Marco teórico fundamental en que se sustenta modelo

Al contrastar estas categorías es posible constatar que en el paradigma lineal causal, el sujeto investigador enfoca y resuelve el control de riesgos o modificación de efectos en personas; desde una perspectiva de sujeto académico que se coloca por fuera de su sociedad y de la naturaleza, mirada que podría definirse como unicultural, antropocéntrica y por sus propios códigos y fundamentación, también como eurocentrada.

En el paradigma empírico ecológico el sujeto se enfoca en la sistemas ecológico-empíricos (*agente, huésped y ambiente*) que se interrelacionan externamente y por cuyo equilibrio debe trabajar. Es el sujeto técnico - académico que busca controlar los desajustes de los sistemas epidemiológicos, no transformar los procesos que subyacen a las expresiones sistémicas, ni transformar la sociedad.

El paradigma de los determinantes sociales es la perspectiva del sujeto institucional, enrolado en el poder público o en las agencias internacionales y que tiene como meta el logro de realizaciones técnicas (tipo metas del milenio) y se propone mejorar una gobernanza, corrigiendo las distorsiones mayores, o estructurales, que amenazan la legitimidad del orden social.

El paradigma etno-social corresponde a una identidad crítica académica, no se plantea el metabolismo sociedad - naturaleza como parte de la determinación. Enfatiza en el poder explicativo de los modos de vida y una etnografía conectada a la reproducción social (movimiento de la cultura del vivir socialmente determinado; destaca la noción probabilística de riesgo en el esquema explicativo; y asume de ese modo como criterio la transformación del patrón de inequidad y de los riesgos.

Por su parte, el paradigma eco-social nace de una perspectiva del ecologismo crítico, motivada por la lucha de grupos sociales que trabajan por la equidad social, de género y etno-cultural; que le define una identidad necesariamente interdisciplinaria e intercultural y un posicionamiento crítico que busca construir demandas para la rendición de cuentas y responsabilidad del poder.

Finalmente, el paradigma de la determinación socialse plantea en la interfase del materialismo crítico, la economía política y la ecología política en su diálogo con una versión crítica de las ciencias de la salud, el ambiente y la sociedad. Se propone descifrar el movimiento de la vida, de su metabolismo histórico en la naturaleza, de los modos de vivir típicos (económicos, políticos, culturales) y del movimiento de los geno-fenotipos humanos, en el marco del movimiento de la materialidad social cuyo eje es la acumulación de capital, nombre que toma en nuestras sociedades la reproducción social.

El núcleo interpretativo de este paradigma es la unidad y diversidad social, ambiental y sanitaria que surgen en medio de la reproducción social y la condición sine qua non de conservar tanto en la interpretación como en la acción de la epidemiología la *unidad* de los procesos alrededor de la acumulacióncomo forma de reproducción frente a la cual puede moverse, en su autonomía relativa, una génesis emancipadora saludable –como diría Samaja– creada por las clases, pueblos y comunidades afectadas por dicha forma de reproducción en su afán por construir un nuevo modelo civilizatorio que implique un metabolismo sociedad naturaleza protector y promotor de la vida.

No hay cabida en el eje vertebrador de este paradigma para colocar como objetivos estratégicos y centrales:ni la redistribución de ingreso y acceso a servicios; ni las medidas limitadas de gobernanza; ni las reivindicaciones aisladas culturales; ni las propuestas de negocio ecológico, es un paradigma que busca acompañar el esfuerzo histórico de los pueblos por derrotar y desmontar el sistema actual de acelerada acumulación de capital, exclusión social y destrucción de la naturaleza, con su modo civilizatorio consumista, contaminante y derrochador. Un paradigma que se piensa y avanza en trance de transformación integral de la sociedad capitalista no sólo para conseguir metas puntuales –convencionalmente medidas en indicadores de *calidad de vida*, como los de ingreso, educativos, y sanitarios- sino para

emancipar a la sociedad y conseguir la vigencia plena de los 4 rubros de la vida: sustentabilidad, soberanía, solidaridad y salud/bioseguridad integral.

Este paradigma sustenta que, sólo en ese marco es posible liberar la economía de su lógica de la muerte y de su esclavitud respecto al valor de cambio para rescatar el profundo valor de uso de las cosas; liberar la política de su papel de dominación y hegemonía para construir una auténtica y autárquica conducción público-social; liberar la cultura de su papel alienante que distorsiona no sólo la educación y expresiones espirituales como el arte, sino aun recursos de la espiritualidad popular como la religión; y finamente liberar al ser humano de ese destructivo antropocentrismo, que le hizo colocar a la madre naturaleza como instrumento poseído en lugar de ser la matriz de la vida.

La epidemiología para tornarse emancipadora, a la par que requirió una revolución metodológica, debió adquirir una identidad que sólo podría ser legítima adhiriendo a la visión profunda de un cambio civilizatorio frente a un sistema social inviable e incompatible con la vida y la salud.

El desafío principal al enfrentar las ideas y métodos de la epidemiología lineal, era evitar el reduccionismo causal-lineal, y hacerlo sin caer en el determinismo mecánico de la totalidad social. La idea de no confundir determinación con determinismo, como lo ha sido explicado ampliamente en otros textos [39]. En efecto, el reto interpretativo era sustituir el pensamiento lineal por el pensamiento complejo, lo que iba de la mano con un reto filosófico y praxiológico.

Era necesario superar una noción enclavada en el pensamiento moderno de los años 70 y 80 de que era inevitable organizar la vida social alrededor de la valorización del valor y juzgar las cosas por su valor de cambio, una herencia ideológica y cultural muy fuerte, filtrada en la lógica de la salud pública funcionalista. Para entender que la salud no es una mercancía, que los elementos que se le relacionan no son mercancías y que es la sociedad la que ha deslindado su valor de uso. Echeverría había comprendido este rasgo del capitalismo con claridad y su relación con la cultura y la ideología:

> "el ethos de la Modernidad capitalista o el ethos del productivismo…centrado en la valorización del valor como forma distorsionante de la reproducción social…..cuyas tendencias ideológico culturales que caracterizan y permiten la reproducción de la modernidad capitalista son: una perspectiva modernista antropocéntrica; un progresismo consumista; el urbanicismo; el economicismo; y el individualismo" [50].

En esta *episteme* –en términos foucaultianos– de la Medicina Social, era necesario rescatar para la salud el concepto de valor de uso, que Marx opone al pensamiento moderno, para hacer "estallar el horizonte de inteligibilidad"[51] de la epidemiología. De ahí surgió la idea de introducir la lógica de la contradicción en el proceso de salud y plantear que en la salud hay una oposición en todos los procesos entre lo que responde al valor de uso y en contraste al valor de cambio, entre lo que nos sirve, protege, da soportey es saludable y lo que daña, deteriora, desmejora y es malsano.

En ese punto nació la idea de las oposiciones en cada dimensión del perfil epidemiológico entre los valores y los contravalores, o mejor, entre el lado saludable y malsano de cada experiencia y condición de nuestras vidas, de la vida de los grupos y de la sociedad en su conjunto. Única manera de conectar de modo inherente, interno, esencial y extendido los efectos en salud con el sistema social dominante, no como algo externo a la vida, sino como algo interno que nos subsume; única manera de superar el pensamiento lineal funcionalista en la salud pública [9].

La determinación social y el replanteamiento hacia una praxis emancipadora

Tal como hemos argumentado, la praxis es el elemento que sustenta y determina la relación objeto-sujeto, y es determinada por estos igualmente. Es en la práctica epidemiológica donde se muestran las profundas implicaciones de los distintos paradigmas y sus construcciones objetivo-subjetivas, pero es además donde se hacen evidentes las proyecciones que benefician o afectan intereses sanitarios y ambientales estratégicos.

Como las sociedades regidas por la acumulación de capital están profundamente escindidas y generan un choque de intereses sociales, la praxis epidemiológica se sitúa siempre en la encrucijada de afectar intereses privados cuando se enrola en la construcción del bien común mayoritario. Para un análisis de la praxis se proponen las siguientes categorías:

- El objeto estratégico de transformación. El recorte del campo de acción, elementos y relaciones básicas de la epidemiología que establece el paradigma.
- Las relaciones sociales y ambientales en el trabajo epidemiológico. La perspectiva desde las que se mira el tipo de relación con sujetos sociales de la población y con la naturaleza.
- El papel de la participación en el conocimiento/incidencia.La forma y grado de involucramiento de los sujetos sociales en el conocimiento / incidencia
- El grado de desarrollo de la acción intersubjetiva / intercultural. Formas y grado de interculturalidad e interdisciplinaridad del quehacer.
- El vínculo histórico de la praxis. El modelo estratégico con el sector social vinculado.

El paradigma lineal de las causas y factores de riesgo al asumir una concepción fraccionaria de la realidad y al sujeto situado por fuera y aparte de la realidad social y la naturaleza, es el caso de una práctica focalizada en dichos factores y graduada de acuerdo a la magnitud y frecuencia de las variables/indicadores que los expresan con sus efectos. El paradigma ecológico empírico amplia el espectro de visión al ambiente pero convertido éste en un campo adicional de variables contextuales.

En correspondencia con la visión positivista de la separación del sujeto respecto a los objetos de la realidad y siguiendo la lógica lineal del orden, la epidemiología positivista separa lo social (x=causa) de lo biológico (y= efecto en los organismos).

Desde este paradigma se asume la lógica de la perspectiva individual del liberalismo que esgrime que los cambios provienen de individuos movilizados por su libre albedrío que se agrupan y finalmente provocan cambios que se expresan en lo colectivo. Desde esta perspectiva la ciencia debe reconocer las características de los individuos, con ellas actuar desde y sobre los individuos; y de ese modo cosechar efectos secundarios positivos en la sociedad. Aquí lo social permanece *invisible*.

En un extremo inverso estará el paradigma determinista colectivo (social mecánico), para el cual el orden social está determinado desde las instancias colectivas de las organizaciones, operando sobre el dominio general, provocando de ese modo efectos en los grupos y finalmente cosechando efectos en los individuos. Desde esta visión el problema radica en reconocer características de las sociedades, sus sistemas de poder; actuar desde y sobre los Estados, instituciones y organizaciones; para cosechar efectos secundarios positivos en los individuos que aparecen como polo pasivo, igualmente invisibilizado. En esta perspectiva se desprecia la contribución de las movilizaciones y operaciones locales o individuales como polo esencial y también generativo del cambio.

Desde el paradigma de los determinantes sociales se coloca en el eje de la praxis la noción de gobernanza definida como "gestión pública participativa y reticular…la puesta en práctica de estilos de gobernar en los que se han difuminado los límites entre los sectores público y privado" [52]. Complementariamente se dice que "alude a un nuevo estilo de gobierno, distinto del modelo de control jerárquico, pero también del mercado, caracterizado por un mayor grado de interacción y de cooperación entre el Estado y los actores no estatales en el interior de redes decisionales mixtas entre lo público y lo privado" [53].

Cabe destacar la intención de superación del control jerárquico o vertical por parte del Estado, y que se rompa con el principio neoliberal del control del mercado. Igualmente la apertura hacia una gestión en salud participativa y *reticular*, así como la tesis de que hay que difuminar los límites entre los sectores públicos y privados como vía para eliminar el autoritarismo oficial.

En estas ideas radican potenciales indicios para una democratización de la acción epidemiológica, sin embargo aprendizajes históricos han enseñado que en contextos de grandes asimetrías en las relaciones de poder, las buenas intenciones y conceptos como estos terminan convertidos en armas de hegemonía, pues quién define a dónde llevan el que se difuminen los límites entre lo público y lo privado o la participación; no son definitivamente las intenciones de actores técnicos quienes lo hacen, sino las fuerzas mayores que subyacen en la política. Ello explica como esto se está convirtiendo en ruta de privatización y debilitamiento del papel rector de lo público, en vehículo de una imposición pública no democrática.

El paradigma eco-social otorga centralidad a la lucha contra la inequidad y busca construir una mejor abogacía y rendición de cuentas a favor de los grupos sometidos a inequidad; trabaja específicamente en las que su autora denomina *rutas de encarnación* de los efectos de lo social en las personas y su biología.

El paradigma de la epidemiología crítica trabaja, en la construcción popular-académica de una sociedad centrada en la vida, la equidad y la plena bioseguridad (socio-biocéntrica), mediante una investigación intercultural, interdisciplinaria y basada en la comunidad, que busca alimentar un proceso de reforma crítica, cuyo eje estratégico es el apoyo desde la ciencia epidemiológica a la defensa de los derechos. Una investigación y monitoreo enfocados en los procesos críticos de la salud, y proyectados hacia le horizonte de una nueva civilización que pueda ser saludable y biosegura, produciendo conocimientos críticos, instrumentos técnicos y herramientas para la rendición de cuentas y control social sobre los grandes dominios de la determinación social general que se expresan en las 4 "S" de la vida (civilización sustentable, soberana, solidaria, saludable/biosegura).

En el marco de este paradigma, se asume como meta fundamental e irrenunciable de la lucha sanitaria la denuncia y proposición de alternativas frente a la acumulación de capital como principio rector, de los sistemas laborales que destruyen masivamente la salud en los espacios de trabajo, de los patrones consumistas que degradan los modos de vivir, y para lograr el control y un giro total de la lógica extractivista y derrochadora de la industria que degrada también la relación sociedad - naturaleza.

No es posible lograr estas metas actuando sobre factores, ni ajustando artificialmente sistemas, ni tampoco buscando mejoras en la gobernanza. Por ello el paradigma de la determinación social es una herramienta para el avance de la justiciabilidad y exigibilidad de derechos sistemáticamente conculcados [54].

La epidemiología crítica contiene por tanto una vocación expresa de derrotar la civilización actual para construir la salud, no remozarla ni pretender tornarla saludable. La epidemiología es pragmáticamente utópica; para serlo tiene que ser en modo teórico y aplicado,

cuantitativa y cualitativa; académica y popular; filosófica, económico política, sociológica, antropológica, histórico geográfica y ecológica; tiene que ser brazo de ruptura y no de gobernanza de la salud pública.

Para ubicar su abordaje o proyección práctica podemos situarlo en relación con los tres elementos del *triángulo de la política* [55]: a) un proyecto emancipador para la salud (teoría crítica sanitaria y proyecto estratégico de incidencia sobre los procesos críticos de la determinación social); b) un bloque social articulado de sujetos-comunidades afectados y movilizados; y c) un cuerpo de conocimientos científicos y herramientas técnicas usadas y rediseñadas en dirección al avance de los dos otros elementos.

Por consiguiente el campo de esta epidemiología crítica es sólo parcialmente académico; asume que ni en los tópicos o problemas más especializados debe incurrirse en el error de pensar que la academia es la única voz cantante, como tampoco puede pensarse que sin un riguroso bagaje científico podemos enfrentar los desafíos de la crítica, defensa y proposición sanitarias [56].

De ahí se desprende, en lo que respecta al papel en las instituciones académicas, las cinco tareas nodales de la universidad crítica: 1) impulso del conocimiento y la investigación crítica; 2) desarrollo de instrumentos técnicos para la operación de cambios hacia la vida saludable; 3) avance de herramientas para el control social, veeduría y rendición de cuentas de los responsables de las políticas y la gestión; 4) consolidación de mecanismos de construcción intercultural e interdisciplinaria de la investigación / incidencia; y 5) con lo anterior, su contribución al empoderamiento democrático sanitario [57].

Para la epidemiología crítica el tema no es la inequidad aislada, sino el cambio integral del sistema social de acumulación/mercado que impide la construcción de las 4 "S" de la vida y la transformación del modelo civilizatorio en los cincos espacios claves donde la reproducción social media la construcción de la salud, o en otros términos, donde se expresan los mecanismos inmediatos de subsunción de la inserción social, con los genotipos y fenotipos de las personas y con los ecosistemas respectivos (figura 9).

Para eso, la epidemiología crítica ha debido modificar su lógica y el arsenal de sus categorías, para poder enfocar, explicar e incidir como una herramienta científica de ruptura, en la construcción de justicia sanitaria en dichas 4 expresiones fundamentales de una vida digna y saludable y los derechos humanos: la liberación respecto al sistema económico-social de acumulación y reproducción de relaciones de poder no soberanas, no solidarias y de profunda inequidad; la liberación respecto de la dominación cultural en todas sus formas interdependientes de género, etno-culturales; la liberación respecto a una construcción destructiva del metabolismo sociedad - naturaleza para conquistar la justicia ambiental y proteger los bienes y derechos de la naturaleza frente a una producción no sustentable, del despojo y de su destrucción.

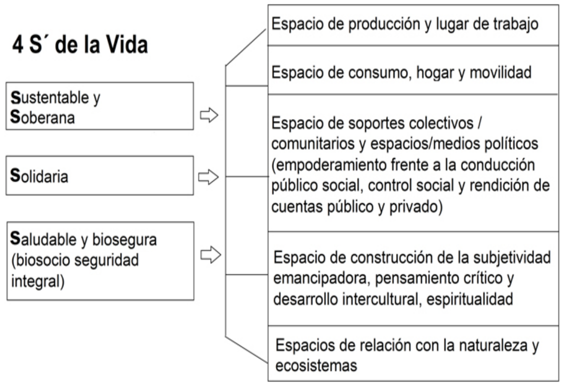

Figura 9. Procesos Críticos en la Determinación del Vivir Saludable. Fuente: Breilh J. Aceleración agro industrial: peligros de la nueva ruralidad del capital, 2011.

La determinación social de la salud y la construcción de una nueva ética para la salud colectiva

En términos más actuales preocupa una asimilación acrítica de categorías de potencial uso epidemiológico como las de *buen vivir; calidad de vida* y *vida saludable*, que han proliferado en los vademécum técnicos o que incluso han sido promovidos a la categoría de principios constitucionales en algunos países. Si no se comprende la necesidad de articularlos a la determinación social de la acumulación de capital terminarán convertidos en simples términos del neo-funcionalismo.

Echeverría nos alerta sobre el error más frecuente de una lectura ligera o desaprensiva acerca de la determinación, sobre la vida espiritual, la cultura y la salud, con su interpretación dialéctica de la determinación de la materialidad social, como también nos informa de los errores del socialismo al concebir la construcción de una materialidad alterna equivocada, reproduciendo la lógica capitalista bajo el Estado socialista. En sus textos encontramos claves importantes para lo que hemos llamado una rebeldía esclarecida para el siglo XXI, para que no se nos filtre un funcionalismo remozado en el *buen vivir* o el *vivir saludable* que defendemos [57].

Una vez en este punto, y habiendo recorrido una panorámica explicación de los fundamentos y requisitos que exige la determinación social, como una herramienta para innovar la gestión, se está en condiciones de esclarecer cuáles son los fundamentos de una nueva ética de la salud. No una bioética, sino una ética integral de la salud que implica la bioética pero mucho más (figura 10).

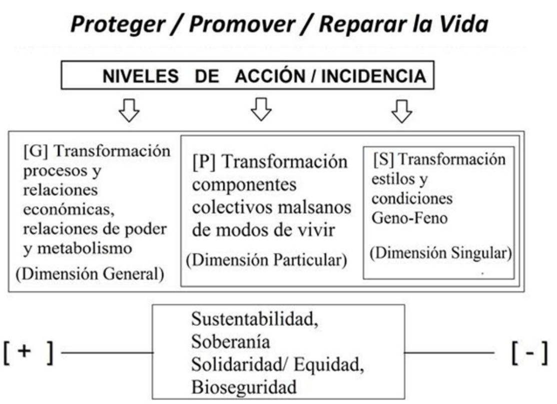

Figura 10. Etica de la salud colectiva

El desarrollo del paradigma esbozado no solamente se proyecta hacia un cambio de la epidemiología sino que también puede apoyar el replanteo de las bases teóricas e instrumentales en el campo de la salud en su dimensión clínica.

Elementos como la definición de casos; construcción de protocolos basados en evidencias; diseño de sistemas de evaluación y monitoreo; la construcción de una práctica clínica intercultural y asimilación de terapias integrativas; el avance de programas de promoción y prevención en los servicios, etc. son apenas algunos ejemplos de cómo el nuevo horizonte de visibilidad para la salud que se abre puede informar el desarrollo de la práctica clínica y armonizar su relación con una práctica epidemiológica igualmente renovada (figura 11).

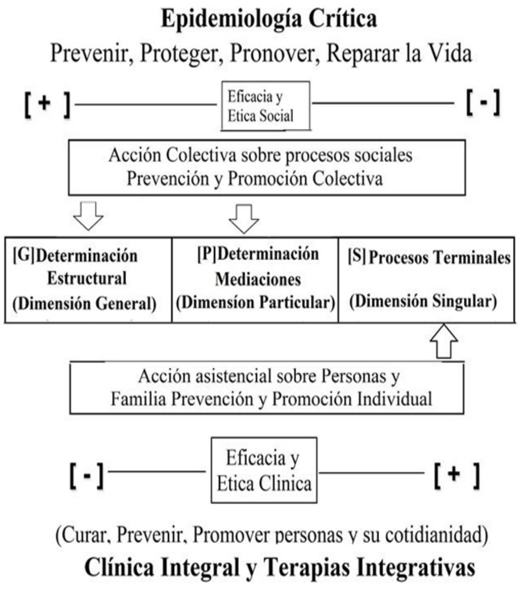

Figura 11. Eficacia y ética social de la epidemiologia crítica

Una vez que hemos recorrido un camino que nos llevó desde las raíces de nuestra propuesta hasta los desarrollos más recientes podemos volver al principio retomando la pregunta ¿qué es en definitiva la epidemiología crítica que proponemos?

La respuesta puede enfocarse e integrarse desde las tres distintas facetas de la salud, en tanto objeto, concepto y campo de acción (figura 12). Sintetizando así: la epidemiología crítica es el conjunto de condiciones, ideas y prácticas/organizaciones que conforman un movimiento, social e históricamente determinado, que llevan a efecto los seres humanos, sea como grupos cohesionados alrededor de los intereses estratégicos de su inserción estructural, filiación cultural y de género, o sea en su condición individual junto con su núcleo familiar, para desentrañar las raíces socio-ambientales de los problemas de salud que genera y reproduce la acumulación, para pensar sobre éstas con un sentido crítico y para actuar en una línea de emancipación respecto a los procesos malsanos que provoca en los órdenes general, particular y singular, en líneas de acción que signifiquen al mismo tiempo una ruptura hacia una sociedad sustentable, soberana, solidaria y saludable/biosegura en todos sus espacios, que hagan posible la preeminencia de procesos protectores y soportes, colectivos, familiares e individuales, que posibiliten el predominio de formas fisiológicas y psíquicas que sustenten una buena calidad de vida biológica y psíquica, posibilitando una mayor longevidad, capacidad de asimilación de noxas, potencialidad para la plena actividad física en todas las edades, disfrute del placer y la espiritualidad.

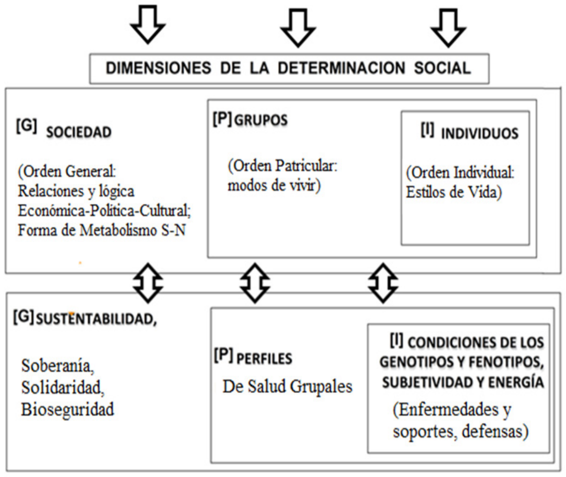

Figura 12. La salud un objeto multidimensional

Agradecimientos

A Carolina Morales, Juan Carlos Eslava y los participantes del seminario interdoctorados *bases teóricas de la determinación social del proceso salud enfermedad*, por sus aportes a un debate esclarecedor; y a Gabriel Jaime Otálvaro C., por su acertado apoyo en la elaboración de este texto.

Referencias

1 Kuhn T. A Estrutura das Revoluções Científicas. 9ª Ed. São Paulo: Perspectiva; 2006. p. 215-257

2 Bourdieu P. O poder simbólico. Rio de Janeiro: Bertrand Brasil; 1998. p.60-61

3 Solórzano A. *¿Fiebre Dorada o Fiebre Amarilla? La Fundación Rockefeller En México.* Guadalajara: Universidad de Guadalajara; 1997.

4 Breilh J. "El vínculo entre producción negligente, débil justicia y ciencia comprada: ¿colusión?" En: Informe de Derechos Humanos Ecuador 2012, ed. Programa Andino de Derechos Humanos. Quito: Universidad Andina Simón Bolívar; 2013.

5 Breilh J. Eugenio Espejo: la otra memoria: nueva lectura de la historia de las ideas científicas. Cuenca: Universidad de Cuenca, Facultad de Ciencias Médicas; 2001.

6 Waitzkin H. Medicine and public health at the end of empire. Boulder: Paradigm Publishers; 2011. p. 9-10.

7 Sigerist H. Civilization and Disease. New York: Cornell University Press; 1944.

8 Rosen G. A history of Public Health. New York: MD Publications; 1958.

9 Breilh J. Crítica a la interpretación capitalista de la epidemiología: un ensayo de desmitificación del proceso salud-enfermedad". México: Universidad Autónoma Metropolitana de Xochimilco; 1977.

10 Laurel AC, Blanco G, Velásquez N, Machetto T, Palomo J, Pérez R, et al. Enfermedad y desarrollo rural: análisis sociológico de la morbilidad de dos pueblos mexicanos. Int J Health Serv 1977;7:401-423

11 Laurell AC. La Salud-enfermedad Como Proceso Social. Revista Latinoamericana de Salud 1982; 2: 7–25.

12 Samaja J. Epistemología y epidemiología. Campinas: Congreso Brasileño de Epidemiología; 1992..

13 Samaja J. Epistemología y metodología: elementos para una teoría de la investigación científica. Buenos Aires: Eudeba; 1993.

14 Almeida-Filho N. Epidemiologia Sem Números: Uma Introduçao Crítica a Ciencia Epidemiológica. Rio de Janeiro: Campus; 1989.

15 Almeida-Filho N. 2000. La Ciencia *Tímida:* Ensayos De Deconstrucción De La Epidemiología. Buenos Aires: Lugar Editorial: Universidad Nacional de Lanus; 2000.

16 Donnangelo C. Saúde e sociedade. São Paulo: Duas Cidades;1979.

17 Tambellini AM. "O trabalho e a doença." En: Guimarães R, organizador. Saúde e medicina no Brasil: contribuçao para um debate. Rio de Janeiro: Edições Graal; 1978. p. 93-119.

18 Ayres R. Sobre o Risco: Para Comprender a Epidemiología. Sao Paulo: HUCITEC; 1997.

19 Victora C, Barros F, Vaughan P. Epidemiologia De La Desigualdad: Un Estudio Longitudinal de 6.000 ninos brasilenos. Washington: OPS; 1992.

20 Victora C, Huttly S, Fuchs S, Olinto M. The Role of Conceptual Frameworks in Epidemiological Analysis: a Hierarchical Approach. Int. J. Epidemiol 1997; 26 (1): 224–227. doi:10.1093/ije/26.1.224.

21 Duarte E. Ciencias Sociales y Salud en América Latina: Tendencias y perspectivas. Montevideo: OPS – CIESU; 1986.

22 Franco S, Nunes E, Breilh J, Laurell AC.. Debates en Medicina Social. Quito: OPS-ALAMES; 1991.

23 Waitzkin H, Iriart C, Estrada A, Lamadrid S. Social medicine in Latin America: productivity and dangers facing the major national groups. Lancet 2001;358(9278):315-23.

24 Waitzkin H, Iriart C, Estrada A, Lamadrid S. Social Medicine Then and Now: Lessons From Latin America. American Journal of Public Health 2001; 91(10): 1592–1601. doi:10.2105/AJPH.91.10.1592.

25 Breilh J. Epidemiología: economía política y salud. 7ª ed. Quito: Corporación Editora Nacional - Universidad Andina Simón Bolívar; 2010. p. 35-44.

26 MacMahon B. Principios y métodos de la epidemiología. México: La Prensa Médica Mexicana; 1975.

27 Rothman KJ. Modern epidemiology. 3ª ed. Filadelfia: Wolters Kluwer Health/Lippincott Williams & Wilkins; 2008.

28 Levins R, Lewontin R. The Dialectical Biologist. Cambridge: Harvard University Press; 1985. p. 1-2.

29 Leavell H, Gurney C. Preventive medicine for the doctor in his community. New York: McGraw-Hill; 1965.

30 Donnangelo C. Aula em Curso de Medicina Social. São Paulo: Associação dos Sociólogos do Estado de São Paulo; 1982.

31 Breilh J. Lo agrario y las 3 's' de la vida. En: Tierra y agua: interrelaciones de un acceso inequitativo. Quito: SIPAE Ediciones; 2010. p. 10-23.

32 Breilh J. Aceleración agroindustrial: peligros de la nueva ruralidad del capital. En: *¿Agroindustria y soberanía alimentaria?: hacia una nueva Ley de Agroindustria y Empleo Agrícola*, 171–190. Quito: SIPAE Ediciones; 2011.

33 Breilh J. Hacia una redefinición de la soberanía agraria: ¿Es posible la soberanía alimentaria sin cambio civilizatorio?. En: Comercialización y soberanía alimentaria, ed. Francisco Hidalgo, PierrilLacroix, and Paola Román, 45–56. Quito: SIPAE Ediciones; 2013.

34 Breilh J. Las Tres 'S' De La Determinación De La Vida: 10 Tesis Hacia Una Visión Crítica De La Determinación Social De La Vida y La Salud. En: Determinação Social Da Saúde e Reforma Sanitária, ed. Roberto Nogueira. Rio de Janeiro: Cebes, Centro Brasileiro de Estudos de Saúde; 2010. p. 87–125

35 Breilh J. Eugenio Espejo: La Otra Memoria: nueva lectura de la historia de las ideas científicas. Cuenca: Universidad de Cuenca, Facultad de Ciencias Médicas; 2001.

36 Breilh J. El Proceso Juliano: pensamiento, utopía y militares solidarios. Cuenca: Corporación Editora Nacional: Universidad Andina Simón Bolívar; 2011.

37 Breilh J, Tillería Y. Aceleración global y despojo en Ecuador: el retroceso del derecho a la salud en la era neoliberal. Quito: Universidad Andina Simón Bolívar; 2009.

38 Marcovic M. Dialéctica de la Praxis. Buenos Aires: Amorrortu Editores; 1972

39 Breilh J. Epidemiología crítica: ciencia emancipadora e interculturalidad. Buenos Aires: Lugar Editorial; 2003.

40 Bradford A. The Environment and Disease. Association or Causation. Proc R Soc Medicine 1965; 58: 295–300.

41 Susser M. Causal Thinking in the Health Sciences. New York: Oxford University Press; 197.3 p. 13-14.

42 Solar, Orielle, and Alec Irwin. 2007. A conceptual framework for action on the social determinants of health. Geneve: Commissionon Social Determinants of Health

43 Timothy E, Whitehead M, Diderichsen F, Bhuiya A, Wirth M. The Social Basis of Disparities in Health. En: Challenging Inequities in Health: From Ethics to Action. New York: Oxford University Press. p. 13–23.

44 Marmot MG, Wilkinson R. Social Determinants of Health. 2ª ed. New York: Oxford University Press; 2006.

45 Breilh J. Las ciencias de la salud pública en la construcción de una prevención profunda." En: Lo biológico y lo social: su articulación en la formación del personal de salud. Washington, D.C.: OPS; 1994. p. 63-100.

46 Krieger N. Epidemiology and the web of causation: Has anyone seen the spider?»Social Science & Medicine 1994; 39(7): 887-903. doi:10.1016/0277-9536(94)90202-X.

47 Krieger N. Theories for social epidemiology in the 21st century: an ecosocial perspective». International Journal of Epidemiology 2001; 30(4): 668–677.

48 Krieger N, Dorling D, McCartney G. Mapping injustice, visualizing equity: Why theory, metaphors and images matter in tackling inequalities. Public Health 2012; 126(3):256-258.

49 Krieger N. Epidemiology and the people's health: theory and context. New York: Oxford University Press; 2012.

50 Echeverría B. Modernidad y Capitalismo (15 Tesis)." Cuadernos Políticos [revista en Internet] 1989; 58: 41–62. [Acceso 12 de julio de 2011]. Disponible en: http://www.cuadernospoliticos.unam.mx/cuadernos/ contenido / CP.58/CP58.41.Bolivar Echeverria.pdf

51 Echeverría B. Valor De Uso y Utopía. México: Siglo XXI Editores; 1998.

52 Natera A. La noción de gobernanza como gestión pública participativa y reticular. Universidad Carlos III de Madrid, Departamento de Ciencia Política y Sociología; 2004. p.5

53 Rhodes R. The New Governance: Governing without Government." Political Studies 1996; 44 (4): 652–667. doi:10.1111/J.1467-9248.1996.tb01747.x.

54 Breilh J. Hacia Una Construcción Emancipadora Del Derecho a La Salud." En *¿Estado Constitucional De Derechos?: Informe Sobre Derechos Humanos, Ecuador 2009*. Quito: Ediciones Abya-Yala; 2010.

55 Matus, Carlos. Adiós Señor Presidente. Santiago: LOM Ediciones; 1998.

56 Breilh J. Hacia una universidad soberana de excelencia y crítica: los principios y caminos de su responsabilidad social." Revista Textos y Contextos 201;7 (12): 39–49.

57 Breilh, Jaime. La subversión del buen vivir (Rebeldía esclarecida para el Siglo XXI). Salud Colectiva 2011;7(3): 389–397.
