# Supplementary material for: Access to health services for chronic disease care during the COVID-19 pandemic in Ecuador: A qualitative analysis using a Social Determinants of Health approach
Source: Glob Public Health. Author manuscript; Available in PMC 2024 Sep 23. (PMC7616628; doi:10.1080/17441692.2023.2224859)
Supplement: S2 [file EMS198626-supplement-S2.pdf]

Sistema de salud de Ecuador

Ruth Lucio, Econ, MSc,[1] Nilhda Villacrés, MD, MSc,[2] Rodrigo Henríquez, MD.[3]

Lucio R,
Villacrés N, Henríquez R.
Sistema de salud de Ecuador.
Salud Publica Mex 2011;53 supl 2:S177-S187.

Resumen

En este trabajo se describen las condiciones de salud en Ecuador y, con mayor detalle, las características del sistema ecuatoriano de salud, incluyendo su estructura y cobertura, sus fuentes de financiamiento, los recursos físicos, materiales y humanos de los que dispone, las tareas de rectoría que desarrolla el Ministerio de Salud Pública, la generación de información en salud, las tareas de investigación, y la participación de los ciudadanos en la operación y evaluación del sistema. También se discuten las innovaciones más recientes que se han implantado en el sistema ecuatoriano de salud dentro de las que destaca la incorporación de un capítulo específico sobre salud a la nueva Constitución que reconoce a la protección de la salud como un derecho humano y la construcción de la Red Pública Integral de Salud.

Palabras clave: sistema de salud; seguridad social; Ecuador

Lucio R,
Villacrés N, Henríquez R.
The health system of Ecuador.
Salud Publica Mex 2011;53 suppl 2:S177-S187.

Abstract

This paper describes the health conditions in Ecuador and, in more detail, the characteristics of the Ecuadorian health system, including its structure and coverage, its financial sources, the physical, material and human resources available, and the stewardship activities developed by the Ministry of Public Health. It also describes the structure and content of its health information system, and the participation of citizens in the operation and evaluation of the health system. The paper ends with a discussion of the most recent policy innovations implemented in the Ecuadorian system, including the incorporation of a chapter on health into the new Constitution which recognizes the protection of health as a human right, and the construction of the Comprehensive Public Health Network.

Key words: health system; social security; Ecuador

(1) Ministerio de Coordinación de Desarrollo Social. Quito, Ecuador.
(2) Instituto Ecuatoriano de Seguridad Social. Quito, Ecuador.
(3) Departamento de Atención Primaria y Cuidado Interdisciplinario, Universidad de Amberes. Amberes, Bélgica.

Fecha de aceptado: 6 de junio de 2011
Solicitud de sobretiros: Ruth Lucio. Ministerio de Coordinación de Desarrollo Social. Av. Amazonas y Santa María, Edificio Tarqui, piso 10. Quito, Ecuador.
Correo electrónico: rlucio@desarrollosocial.gob.ec

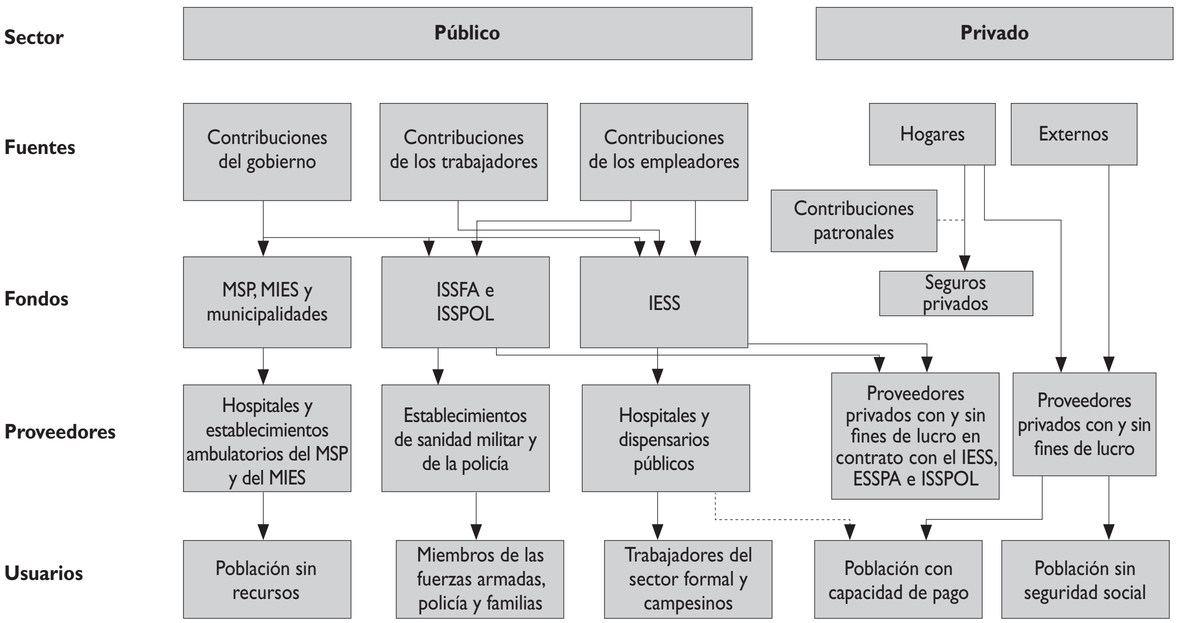

MSP: Ministerio de Salud Pública
MIES: Ministerio de Inclusión Económica y Social
ISSFA: Instituto de Seguridad Social de las Fuerzas Armadas
ISSPOL: Instituto de Seguridad Social de la Policía Nacional
IESS: Instituto Ecuatoriano de Seguridad Social

El sistema de salud de Ecuador está compuesto por dos sectores: público y privado. El sector público comprende al Ministerio de Salud Pública (MSP), el Ministerio de Inclusión Económica y Social (MIES), los servicios de salud de las municipalidades y las instituciones de seguridad social (Instituto Ecuatoriano de Seguridad Social, Instituto de Seguridad Social de las Fuerzas Armadas e Instituto de Seguridad Social de la Policía Nacional). El MSP ofrece servicios de atención de salud a toda la población. El MIES y las municipalidades cuentan con programas y establecimientos de salud en los que también brindan atención a la población no asegurada. Las instituciones de seguridad social cubren a la población asalariada afiliada. El sector privado comprende entidades con fines de lucro (hospitales, clínicas, dispensarios, consultorios, farmacias y empresas de medicina prepagada) y organizaciones no lucrativas de la sociedad civil y de servicio social. Los seguros privados y empresas de medicina prepagada cubren aproximadamente a 3% de la población perteneciente a estratos de ingresos medios y altos. Además existen al menos 10 000 consultorios médicos particulares, en general dotados de infraestructura y tecnología elementales, ubicados en las principales ciudades y en los que la población suele hacer pagos directos de bolsillo en el momento de recibir la atención.

Contexto

Demografía

Según proyecciones del Instituto Nacional de Estadística y Censos (INEC), Ecuador cuenta con 14.2 millones de habitantes, 50.1% son hombres y 49.9% mujeres. Alrededor de 66% de la población total del país es urbana y 33% reside en las cinco ciudades más importantes del país.[1]

Las tendencias de crecimiento de población han cambiado debido a factores como la reducción de la tasa bruta de natalidad de 32.4 a 11.4 nacimientos por 1 000 habitantes entre 1981 y 2010, la migración hacia Europa y Estados Unidos, y la disminución de la tasa de mortalidad de 6.7 muertes por 1000 habitantes en 1981 a 4.3 en 2008.[2]

La estructura de la población por grupos de edad aún tiene la forma característica de los países en desarrollo. Sin embargo, durante la década pasada se registró una disminución de la base de niños y jóvenes, con ensanchamiento de la punta de la pirámide poblacional por envejecimiento de la población. Entre 1990 y 2009 aumentó la población de 15 a 64 años (de 56.9 a a 63.1% de la población total) y de 65 años y más (de 4.3 a 6.2%), en detrimento de la población de entre 0 y 14 años de edad (de 38.8 a 30.7%).[3]

Condiciones de salud

En 2008 las enfermedades crónicas no transmisibles (diabetes mellitus, enfermedades cerebrovasculares, enfermedades hipertensivas, cardiopatía isquémica,

Cuadro I
Indicadores básicos, Ecuador, 2010

Población total	14 204 900
Esperanza de vida al nacer	75
Tasa de mortalidad general (2008)	4.35
Tasa de mortalidad infantil (2009)	11.4
Razón de mortalidad materna (2008)	80
Prevalencia de hipertensión arterial	28.7
Porcentaje de partos atendidos por personal calificado (2005)	80
Cobertura de vacunación en menores de 1 año (%) (2008)	
Tuberculosis (BCG)	99
Sarampión	97.6
Población con seguridad social	4 036 300
Población sin seguridad social	10 168 600
Gasto total en salud como porcentaje del PIB (2008)	5.9
Gasto per cápita en salud* (2008)	474
Gasto público en salud como porcentaje del gasto total en salud (2008)	39.5

Fuente: referencias 1-7
* Dólares PPA (paridad de poder adquisitivo)

insuficiencia cardíaca y cirrosis), los accidentes de transporte terrestre y las agresiones fueron las principales causas de muerte en la población general de Ecuador. La influenza y la neumonía son las únicas enfermedades transmisibles que figuraron entre las diez principales causas de muerte.[2] Esto refleja un proceso de transición epidemiológica donde coexisten los problemas crónicos no trasmisibles y las enfermedades transmisibles. El cuadro en mujeres era similar al nacional (cuadro II). Las cuatro primeras causas de mortalidad eran la diabetes mellitus, las enfermedades hipertensivas, las enfermedades cerebro-vasculares y la influenza y neumonía, responsables de 25% de las muertes totales. En el caso de los hombres, las principales causas de muerte fueron los accidentes de transporte terrestre y las agresiones, responsables de 12% de las muertes totales (cuadro III). A estas causas le siguieron las enfermedades isquémicas del corazón, los padecimientos cerebro-vasculares, la influenza y neumonía, las enfermedades hipertensivas y la diabetes mellitus, que provocaron alrededor de la cuarta parte de las muertes totales.

La tasa de mortalidad infantil en 2009 fue de 20 por 1 000 nacidos vivos.[6] La primera causa de muerte en menores de un año fueron los trastornos relacionados con duración corta de la gestación y con bajo peso al nacer no clasificados en otra parte, lo que hace pensar en un control deficiente del embarazo, entre otros factores determinantes. Le siguen la neumonía, la sepsis bacteriana, la dificultad respiratoria y la neumonía congénita.[2]

En cuanto a muertes maternas, la razón de mortalidad fue de 140 por 100 000 nacidos vivos.[6] La hemorragia posparto es la principal causa de muerte, seguida por los trastornos hipertensivos durante el embarazo (preeclampsia, eclampsia), las anormalidades de la

Cuadro II
Diez principales causas de muerte en mujeres, Ecuador, 2008

Enfermedad	*Número*	*Tasa**	*%*
1. Diabetes mellitus	1 931	28.0	7.6
2. Enfermedades cerebro-vasculares	1 685	24.5	6.6
3. Enfermedades hipertensivas	1 580	22.9	6.2
4. Influenza y neumonía	1 521	22.1	6.0
5. Insuficiencia cardiaca	1 183	17.2	4.6
6. Enfermedades isquémicas del corazón	1 056	15.3	4.1
7. Enfermedades del sistema urinario	933	12.0	3.2
8. Neoplasia maligna del estómago	719	10.4	2.8
9. Neoplasia maligna del útero	708	10.3	2.8
10. Cirrosis y otras enfermedades del hígado	665	9.7	2.6

* Tasa por 100 000 mujeres
Fuente: Referencia 2

Cuadro III
Diez principales causas de muerte en hombres, Ecuador, 2008

Enfermedad	*Número*	*Tasa**	%
1. Agresiones	2 279	32.9	6.6
2. Accidentes de transporte terrestre	2 188	31.6	6.3
3. Enfermedades cerebrovasculares	1 723	24.9	5.0
4. Enfermedades isquémicas del corazón	1 704	24.6	4.9
5. Enfermedades hipertensivas	1 685	24.4	4.9
6. Influenza y neumonía	1 666	24.1	4.8
7. Diabetes mellitus	1 579	22.8	4.6
8. Insuficiencia cardiaca	1 134	16.4	3.3
9. Cirrosis y otras enfermedades del hígado	1 127	16.3	3.3
10. Neoplasia maligna del estómago	945	13.7	2.7

* Tasa por 100 000 hombres
Fuente: Referencia 2

dinámica del trabajo de parto, la sepsis puerperal, el embarazo ectópico y el aborto no especificado.[2]

Años de vida saludable perdidos

La mortalidad es un indicador útil, pero tiene el inconveniente de que no incorpora todos los daños a la salud que se producen en una sociedad. Por esta razón se han diseñado indicadores como los años de vida saludable (AVISA) perdidos que contabilizan los años de vida saludable que se pierden tanto por muerte prematura como por discapacidad. El Centro de Estudios de Población y Desarrollo Social publicó este indicador en 1999 y desagregado por provincias en 2000.[8,9] Los AVISA perdidos, tanto por muerte prematura como por discapacidad, sumaron 2 143 353; 58.9% se concentraron en hombres y el resto en mujeres. Las enfermedades transmisibles, de la nutrición y de la reproducción concentraron 33.7% del total, las enfermedades no transmisibles 42.2% y las lesiones 24.1 por ciento. En 2008, de acuerdo con la Organización Mundial de la Salud (OMS), las enfermedades no transmisibles concentraron 45% de los AVISA perdidos, 30% las enfermedades no transmisibles y 25% las lesiones.[6]

Estructura y cobertura

El sistema de salud de Ecuador está compuesto por dos sectores, público y privado.[10] El sector público comprende al Ministerio de Salud Pública (MSP), el Ministerio de Inclusión Económica y Social (MIES), los servicios de salud de las municipalidades y las instituciones de seguridad social [Instituto Ecuatoriano de Seguridad Social (IESS), Instituto de Seguridad Social de las Fuerzas Armadas (ISSFA) e Instituto de Seguridad Social de la Policía Nacional (ISSPOL)]. El MSP ofrece servicios de atención de salud a toda la población. El MIES y las municipalidades cuentan con programas y establecimientos de salud en los que también brindan atención a la población no asegurada. Las instituciones de seguridad social cubren a la población asalariada afiliada. El sector privado comprende entidades con fines de lucro (hospitales, clínicas, dispensarios, consultorios, farmacias y empresas de medicina prepagada) y organizaciones no lucrativas de la sociedad civil y de servicio social. Los seguros privados y empresas de medicina prepagada cubren aproximadamente a 3% de la población perteneciente a estratos de ingresos medios y altos. Además, existen cerca de 10 000 consultorios médicos particulares, en general dotados de infraestructura y tecnología elementales, ubicados en las principales ciudades y en los que la población suele hacer pagos directos de bolsillo en el momento de recibir la atención.

¿Quiénes son los beneficiarios?

La nueva Constitución de 2008 señala que "la salud es un derecho que garantiza el Estado, cuya realización se vincula al ejercicio de otros derechos, entre ellos el

Diez hitos en la historia del sistema ecuatoriano de salud

1935	Establecimiento del Servicio Médico del Seguro Social como sección del Instituto Nacional de Previsión (actual Instituto Ecuatoriano de Seguridad Social)
1951	Creación de la Sociedad de Lucha Contra el Cáncer
1967	Creación del Ministerio de Salud Pública
1980	Creación del Consejo Nacional de Salud
1994	Promulgación de la Ley de Maternidad Gratuita
1998	Reforma constitucional: nueva sección específica sobre salud
2001	Promulgación de la Ley sobre Seguridad Social
2006	Creación del Programa de Aseguramiento Universal en Salud
2008	Incorporación de un capítulo específico sobre salud a la nueva Constitución que reconoce a la protección de la salud como un derecho humano
2009	Inicio de la construcción de la propuesta de Red Pública Integral de Salud a partir de su incorporación en la nueva Constitución

derecho al agua, alimentación, nutrición, educación, cultura física, trabajo, seguridad social, ambientes sanos y otros que sustentan el buen vivir. El Estado garantizará este derecho mediante políticas económicas, sociales, culturales, educativas y ambientales; y el acceso permanente, oportuno y sin exclusión a programas, acciones y servicios de promoción y atención integral de salud, salud sexual y salud reproductiva. La prestación de los servicios de salud se regirá por los principios de equidad, universalidad, solidaridad, interculturalidad, calidad, eficiencia, eficacia, precaución y bioética, con enfoque de género y generacional".[11]

El MSP, con la provisión más amplia de servicios del país, 47% de unidades ambulatorias y hospitalarias, y los hospitales de referencia nacional más grandes del país, cubre alrededor del 51% de la población ecuatoriana.[12]

Los trabajadores del sector formal de la economía, incluyendo al sector campesino, tienen derecho a afiliarse al IESS. Este instituto cubre por lo menos a 20% del total de la población ecuatoriana (cuadro IV).[13]

El ISSFA y el ISSPOL cubren a poco más de 5% de la población con seguros públicos, que representan 2% de la PEA.[14] La cobertura de salud de estos sistemas es familiar e incluye a los hijos hasta los 25 años de edad.

En resumen, si bien la ley proclama la cobertura universal en salud, en los hechos todavía existe una limitada cobertura real con servicios cuya calidad no siempre es la óptima.[15]

En 1994 se promulgó la Ley de Maternidad Gratuita (LMG), que se reformó en 1998 constituyéndose en un seguro de salud que garantiza a las mujeres atención a la salud gratuita y de calidad durante su embarazo, parto y posparto, así como acceso a programas de salud sexual y reproductiva.[17] Esta ley también ampara la atención a la salud a los recién nacidos y los menores de 5 años como una acción de salud pública gratuita responsabilidad del Estado. En 2007 el MSP asumió la LMG como un programa regular.[18]

El Bono de Desarrollo Humano (BDH) es una transferencia monetaria mensual que otorga el gobierno a las personas que se encuentran en situación de pobreza.[19,20] Los beneficiarios son familias que se ubican dentro de los dos quintiles de ingresos más bajos de acuerdo con el índice de bienestar del Sistema de Selección de Beneficiarios.[21] Este sistema fija el nivel de recursos de las familias tomando en consideración la composición demográfica del hogar, sus activos y otras variables. En familias con hijos en edad escolar el bono está condicionado al cumplimiento de requisitos establecidos por el Programa de Protección Social, que incluyen metas de atención de salud y asistencia a establecimientos

Cuadro IV
Cobertura de la seguridad social, Ecuador 2010

Seguro	*Cotizantes*	*Jubilados*	*Montepío (viudez y orfandad)*	*Dependientes*	*Total*	*%*
IESS	1 826 911	187 911	90 060	545 685	2 650 567	65.7
SSC	227 694	34 729	-	689 723	952 146	23.6
ISSFA	37 719	25 379	11 576	148 384	223 058	5.5
ISSPOL	39 286	10 450	6 616	154 179	210 531	5.2
Total	2 131 610	258 469	108 252	1 537 971	4 036 302	100

Fuente: Referencia 16

de educación. El objetivo del programa en salud es contribuir a la disminución de la desnutrición crónica y enfermedades prevenibles en los menores de 5 años.

En 2007 surgió el Programa de Cobertura de Enfermedades Catastróficas, ejecutado mediante la Red de Protección Solidaria integrada por el MIES y MSP. Este programa financia todos los costos relacionados al tratamiento de enfermedades de alto costo en casos seleccionados. En 2010 se destinó a este programa un presupuesto cercano a los 60 millones de dólares.[22]

¿En qué consisten los beneficios?

Los ecuatorianos que mayores beneficios en salud reciben son aquellos que están cubiertos por alguno de los institutos de seguridad social. Los beneficiarios del IESS están protegidos contra la contingencia de enfermedad por las siguientes prestaciones: asistencia médica, quirúrgica, dental y farmacológica integral. Estas prestaciones incluyen consulta externa, urgencias, medicina curativa, preventiva y de rehabilitación mediante la provisión de ortesis y prótesis; atención quirúrgica y hospitalización; atención médica domiciliaria; subsidio en dinero por enfermedad, y atención en unidades médicas ajenas al IESS. Los beneficiarios del IESS también reciben compensación de gastos médicos y atención médica mediante convenios suscritos con diversas clínicas privadas.[23]

El ISSFA ofrece a sus miembros servicios de consulta externa, emergencia, hospitalización y medicina ambulatoria para pacientes aquejados por padecimientos crónicos graves. El titular tiene una cobertura de 100%, mientras que sus dependientes, excluyendo a los padres, tienen cobertura sólo para ciertas enfermedades. El ISSPOL otorga servicios de asistencia médica, quirúrgica, obstétrica, odontológica, de hospitalización y farmacéutica, prótesis, ortesis y rehabilitación, así como acciones de medicina preventiva.[24,25,26]

La LMG, antes de ser un programa regular del MSP, cubría por separado los gastos en medicinas, insumos, micronutrientes, suministros, exámenes básicos de laboratorio y exámenes complementarios para la atención de las mujeres embarazadas, los recién nacidos y los menores de 5 años de edad.[18] En la actualidad complementa las prestaciones regulares del MSP.

El BDH brinda, desde agosto de 2009, el subsidio directo más grande que otorga el gobierno a la población pobre (Q1, Q2) dentro del sector social, que asciende a 35 dólares mensuales al jefe de familia. En 2011 el presupuesto estimado para este programa bordea los 500 millones de dólares. El programa de enfermedades catastróficas atiende casos con un tope de hasta 100 000 dólares luego de ser analizados y aprobados por un comité técnico, El costo promedio por caso en 2009 fue de 9 600 dólares. De mantenerse al 2011 el presupuesto ejecutado durante 2010, el programa podría llegar a atender alrededor de 6 000 casos.

Financiamiento

¿Quién paga?

La Constitución en el artículo 366 estipula lo siguiente: "El financiamiento público en salud será oportuno, regular y suficiente, y deberá provenir de fuentes permanentes del Presupuesto General del Estado. Los recursos públicos serán distribuidos con base en criterios de población y en las necesidades de salud."[11]

Según la Ley de Seguridad Social 2001, el Seguro General Obligatorio (SGO) del IESS tiene tres fuentes de financiamiento: la aportación individual obligatoria de los afiliados, la aportación obligatoria de los empleadores públicos o privados y la contribución del Estado.[27] Señala también que los servicios de salud y prestaciones del Seguro Social Campesino (SSC) se financian con recursos provenientes de las siguientes fuentes: el aporte solidario de los empleadores, el aporte de los afiliados al SGO, la contribución obligatoria de seguros públicos y privados, el aporte diferenciado de familias protegidas por el SSC, la contribución del Estado y las asignaciones suplementarias determinadas por el Poder Ejecutivo.

El ISSPOL se financia con un aporte de los miembros de la policía en servicio activo de 2.5% de su salario y un aporte del patrono (Estado) de 3 por ciento. Es el único caso en el país donde también los retirados aportan 2.5% de sus pensiones.[25]

En el caso del ISSFA, los recursos provienen de los aportes de los militares en servicio activo (equivalente al 3.35% de su haber militar) y los aportes patronales que hace el Ministerio de Defensa (5.85% del haber militar del personal en servicio activo y 0.52% del 2% del haber militar de un soldado en servicio activo, multiplicado por el número de aspirantes a oficiales, tropa y conscriptos).[24,26]

¿A cuánto asciende el gasto en salud?

El gasto en salud en Ecuador ha crecido consistentemente en los últimos 10 años.[7] De 4.2% del PIB pasó a 5.9% en 2008. El gasto per cápita en salud aumentó de 202 dólares en 2000 a 474 dólares en 2008. El gasto público se incrementó de 31.2% del gasto total en salud a 39.5 por ciento. El gasto privado en salud en 2008 representó 60.5% del gasto total.[7] El grueso de este gasto es de bolsillo. El gasto en seguros privados representó sólo 4.8% del gasto privado total en salud.

¿Quién reúne el dinero y cómo lo distribuye?

El gobierno es el encargado de establecer los criterios de distribución de recursos. El organismo ejecutor es el Ministerio de Finanzas (MF), responsable de la recolección y distribución de los recursos fiscales, esto es, impuestos, ingresos del petróleo, préstamos y donaciones de organismos multilaterales y organizaciones no gubernamentales. El MF se encarga de la aprobación final de los presupuestos de las entidades adscritas y empresas del Estado. También es responsable de la planificación de los recursos financieros del gobierno nacional. Además del MSP, las instituciones de seguridad pública regidas por el gobierno (ISSPOL e ISSFA) deben someterse a las disposiciones del MF.

El IESS fija y maneja su presupuesto de manera autónoma, con la aprobación del Consejo Directivo de la institución.

Recursos

¿Con qué infraestructura y equipo se prestan los servicios de salud?

Ecuador tiene registrados 3 847 establecimientos de atención a la salud, de los cuales 80% pertenecen al sector público.[28] El MSP concentra 47% de las unidades y el IESS otro 24 por ciento. Una pequeña proporción (10%) pertenece a municipios, ministerios diferentes al MSP, ISSFA, ISSPOL y otras entidades. El sector privado concentra alrededor del 20% de los establecimientos de salud, 14% en instituciones con fines de lucro y el restante 6% en organismos que ofrecen atención a la salud sin fines de lucro.

La mayor parte de los establecimientos con internación registrados en 2007 (545) eran clínicas particulares, que representan 75% del total.[28] Le siguen en importancia los hospitales cantonales, los hospitales generales y, en una mínima proporción, los hospitales de especialidad. La mayor parte de los establecimientos sin internación que se ubican en las áreas urbanas son dispensarios médicos. En las zonas rurales, la mayor proporción corresponde a subcentros de salud, a los que siguen los dispensarios médicos.

El MSP cuenta con 1 674 establecimientos de salud ambulatorios y 125 hospitales: 28 hospitales generales, 79 hospitales cantonales y 18 hospitales de especialidad.[28] El IESS brinda atención en 902 unidades ambulatorias entre servicios y anexos en las empresas, además de 18 centros hospitalarios, tres regionales y 15 provinciales. El ISSFA dispone de 72 establecimientos con servicios de atención ambulatoria y 16 de hospitalización. El ISSPOL cuenta con 35 unidades operativas, de las cuales dos son hospitalarias.

La Sociedad de Lucha contra el Cáncer, entidad privada sin fines de lucro que cubre buena parte de la demanda nacional de diagnóstico y tratamiento del cáncer, ofrece servicios en instituciones regionales con autonomía administrativa y financiera ubicadas en las principales ciudades del país y cuenta con ocho hospitales especializados y cuatro centros de atención sin internación.

En 2008 el sistema ecuatoriano de salud contaba con 21 575 camas (10 273 privadas y 11 302 públicas), que arrojan una razón de 1.6 camas por 1 000 habitantes.[29]

De acuerdo con el INEC, en 2005 Ecuador contaba con 53 mamógrafos, 48 tomógrafos y 15 equipos de resonancia magnética, cifras que arrojan razones de 3.9 mamógrafos, 3.5 tomógrafos y 1.1 equipos de resonancia magnética por millón de habitantes, respectivamente. En el periodo 2008-2010 el gobierno ecuatoriano compró 42 mamógramos y 23 tomógrafos, con lo cual mejoró sustancialmente las razones de estos equipos.[30]

¿Con qué recursos humanos se prestan los servicios?

Aunque entre 1996 y 2010 se incrementó la cantidad de recursos humanos empleados dentro del sistema de salud en aproximadamente 21%, en Ecuador todavía prevalece un déficit relativo de médicos y enfermeras, que se agrava por un sistema de contratación laboral en el sector público de cuatro y seis horas.

En 2007 Ecuador contaba con alrededor de 75 mil trabajadores de la salud.[28] La razón de médicos por 1000 habitantes era de 1.9, muy inferior a la países como Argentina (3.0) y Uruguay (3.6). La tasa de enfermeras por 1 000 habitantes a nivel nacional era de 0.6, que está por debajo del promedio latinoamericano, que en 2000 era de 0.8. El grueso de los médicos y enfermeras trabajan en el MSP y no se tiene información precisa sobre el número de médicos y enfermeras que trabajan en el sector privado. La tendencia institucional de contratación de recursos humanos para 2010 parecer haber cambiado, ya que el IESS se encuentra realizando contrataciones de manera importante para cubrir el incremento de cobertura aprobado.

Los médicos tienden a concentrarse en las grandes ciudades, lo que genera problemas serios de distribución. En la provincia de Pichincha hay 2 médicos por 1 000 habitantes, mientras que en las provincias de Galápagos y Orellana esta razón es de apenas 0.56 y 0.43, respectivamente.[28]

¿Cuánto se gasta en medicamentos y cuál es el nivel de acceso a ellos?

En 2004 el mercado farmacéutico ecuatoriano alcanzó ventas por un valor aproximado de 556 millones de dóla-

res, lo que representó casi 2% del PIB.[31] La participación del sector privado fue de 88.2% mientras que el sector público sólo participó con 11.8% del total. El valor del gasto en medicamentos per cápita fue de alrededor de 18 dólares por año. En el año 2007 el ingreso del sector farmacéutico ascendió a 930.3 millones de dólares, que representan 2.03% del PIB.

En 2000 se aprobó la Ley de Medicamentos Genéricos, que obliga a las instituciones públicas a emplear fármacos intercambiables y equivalentes a los patentados. Esta Ley también reduce el margen de beneficios de las farmacias con respecto a los medicamentos patentados y las alienta a dispensar versiones genéricas.[32]

La Comisión Nacional de Medicamentos e Insumos del Consejo Nacional de Salud (CONASA) actualiza, publica y difunde bianualmente el cuadro nacional de medicamentos básicos. Actualmente se acaba de aprobar la octava revisión publicada, que incluye una lista de medicamentos esenciales de acuerdo con la definición de la OMS.[33,34]

Las medicinas se comercializan en un mercado regulado con un esquema de fijación de precios que incluye diversos rubros que inciden de manera problemática en el costo final del producto.

Desde 2007 Ecuador ha impulsado medidas para dar cumplimiento al derecho a la salud promoviendo activamente acuerdos en foros internacionales y llevando a cabo negociaciones regionales de precios de los medicamentos antirretrovirales empleados para el tratamiento del VIH/SIDA. También ha establecido acuerdos bilaterales con productores de biológicos como Cuba e impulsado la producción nacional de estos insumos a través de una empresa pública recientemente creada (Enfarma) y de laboratorios radicados en el país. A través de la nueva ley de compras públicas ha promovido asimismo subastas voluminosas para abaratar ofertas con miras a un futuro acuerdo de compra global entre las instituciones públicas.

¿Quién genera la información y quien produce la investigación?

La mayor parte de la información oficial la genera el INEC, cuya misión es generar y difundir información estadística (en el caso del sector salud respecto a hechos vitales y servicios de salud) del país con el propósito de facilitar la evaluación del desarrollo de la sociedad y la economía. El Banco Central del Ecuador, como responsable de generar las cifras correspondientes a las cuentas nacionales, genera también información valiosa respecto a los principales índices macroeconómicos sobre gasto en salud.

En la Ley Orgánica del Sistema Nacional de Salud (LOSNS) de 2002 se establece que el MSP, con el apoyo del CONASA, es responsable de impulsar una política de investigación orientada a las prioridades nacionales y al desarrollo y transferencia de tecnología, teniendo en cuenta la realidad del país, para lo cual debe mantener un enfoque pluricultural que incluya la promoción y el desarrollo de la medicina tradicional.[36]

Una referencia estandarizada sobre la situación de la investigación científica es el número de profesionales que obtienen títulos académicos de posgrado. En Ecuador esta cifra llega apenas a 300. FUNDACYT, un organismo privado, presidido por el Secretario Nacional de Ciencia y Tecnología, ha financiado proyectos de muy distinta índole, desde iniciativas de biomedicina hasta el desarrollo de laboratorios para la aplicación de biotecnología. Aunque la mayor parte de los esfuerzos provienen de personas o instituciones aisladas y su objetivo es la satisfacción de una necesidad puntual, en 2007 se financiaron 438 proyectos de investigación, fundamentalmente provenientes de universidades.[36]

Rectoría

¿Quién pone orden en el sector salud y cómo se regula la atención?

La Constitución enfatiza fuertemente la necesidad de recuperar la regulación para el Estado ecuatoriano y señala que al MSP corresponde el ejercicio de la rectoría en salud, así como la responsabilidad de la aplicación, control y vigilancia del cumplimiento de la LOSNS y las normas dictadas para su vigencia.

El MSP tiene una Dirección de Salud en cada provincia y en su interior áreas de salud que son circunscripciones geográfico-poblacionales. Estas áreas funcionan como unidades de desconcentración programática, administrativa y presupuestaria de las cuales dependen los servicios básicos de salud.

A partir de 2010, con la emisión de la nueva estructura territorial ecuatoriana, se está produciendo una reorganización que tendría coincidencia territorial al menos para los ámbitos de salud, educación e inclusión social. Se está estructurando el país en regiones, provincias, distritos y circuitos.

El MSP también ejerce la rectoría de las entidades de salud mediante el otorgamiento del permiso de funcionamiento a las empresas de salud tanto públicas como privadas y de medicina prepagada. Además del MSP, varias entidades participan en la supervisión y regulación, de acuerdo con su ámbito de competencia, de la actividad de las diferentes instituciones que componen el sistema de salud de Ecuador. La Contraloría General del Estado se encarga, en el ámbito financiero, de controlar los hospitales y las unidades médicas, y

examina y evalúa la gestión financiera, administrativa y operativa de entidades donde el Estado tiene participación. La Superintendencia de Compañías autoriza la constitución de entidades de medicina prepagada y la Superintendencia de Bancos y Seguros (SBS) ejerce el control sobre las unidades médicas del IESS conforme a la Ley de Seguridad Social. La inclusión de la SBS como organismo de control dentro del sistema de salud es producto de la Ley de Seguridad Social de 2001.

¿Quién vigila las actividades que impactan la salud?

Una de las funciones del MSP es normar, regular y controlar las actividades realizadas por entidades públicas y privadas que se vinculan con la salud de las personas y el medio ambiente.

En la atención de salud son poco estructurados todavía los controles del MSP. En el caso del IESS se ha emprendido en el último año un interesante proceso de fortalecimiento de auditoría técnica previo a los pagos. ISSFA e ISSPOL, por su parte, cuentan con sistemas de auditoría técnica desde hace varios años, lo cual les ha permitido mantener la calidad del servicio y contener costos.

¿Quién evalúa?

No existe un organismo encargado de llevar a cabo una evaluación coordinada del sistema de salud de Ecuador. Estas funciones las cumplen de manera independiente varias instituciones relacionadas con la salud que se han ido sumando a esta función a partir de cambios en las leyes o la creación de nuevos organismos orientados por los intentos de reformas. Por ejemplo, desde 2001, la SBS ha sido responsable de la supervisión de las instituciones de la seguridad social. Para ello la ley establece que empresas especializadas realicen auditorías médicas a los prestadores de salud bajo la supervisión de la SBS.

No obstante, el MSP es el principal agente de evaluación del sistema, tanto por su carácter de rector del sector salud como por las disposiciones de la Ley de Transparencia de 2004, la cual obliga a todas las instituciones públicas a difundir información sobre sus funciones.[37] Dicha ley define mecanismos de rendición de cuentas tales como metas e informes de gestión e indicadores de desempeño. El MSP difunde información en este sentido a través de su página en internet mediante la publicación de una tabla que resume ciertos indicadores de salud. En ella se dan cifras respecto a las metas establecidas para el año y se confrontan con los valores reales y los porcentajes de cumplimiento.

El actual gobierno (2007-2011) creó el Ministerio de Coordinación de Desarrollo Social, cuya función es la de concertar políticas y acciones de los ministerios del área social, entre los que se encuentra el MSP. Uno de los objetivos es realizar un monitoreo regular de los programas prioritarios de dicha área mediante la definición de una línea de base y la evaluación de resultados en indicadores de cobertura, calidad y eficiencia.

Voz de los usuarios

¿Cómo participan los ciudadanos en la gestión y evaluación del sistema?

En la Ley Orgánica de Salud de 2006, dentro del capítulo sobre derechos y deberes que las personas y el Estado tienen en relación con la salud, se declara que uno de ellos es "participar de manera individual o colectiva en las actividades de salud y vigilar el cumplimiento de las acciones en salud y la calidad de los servicios, mediante la conformación de veedurías ciudadanas u otros mecanismos de participación social; y ser informado sobre las medidas de prevención y mitigación de las amenazas y situaciones de vulnerabilidad que pongan en riesgo su vida."

Un organismo de control ciudadano es la Comisión de Control Cívico de la Corrupción (CCCC), creada en 1997 e institucionalizada en la reforma constitucional de 1998 como uno de los órganos de control del Estado, con autonomía e independencia económica, política y administrativa. El CCCC se encarga de recibir, tramitar e investigar denuncias ciudadanas sobre posibles actos de corrupción. Cuando se encuentran indicios de responsabilidad, pone sus conclusiones en conocimiento del Ministerio Público, la Contraloría General del Estado o el órgano jurisdiccional competente de conformidad con la Ley.

Un instrumento que coordina este organismo es el de las "veedurías ciudadanas", que son grupos de ciudadanos que participan en acciones de control social. Sus actividades se centran básicamente en la vigilancia de las actividades públicas, en la auditoria a los sectores de la salud, educación, medio ambiente y vivienda, y en la prestación de servicios públicos domiciliarios.

Innovaciones

¿Qué innovaciones recientes se han implantado?

En 2001 se aprobó la Ley de Seguridad Social, que introdujo nuevos elementos, apartando la provisión de servicios de salud de las prestaciones de seguridad social generales y estableciendo mecanismos de control al sector. Esta ley se encuentra vigente todavía, aunque en la actualidad el gobierno se encuentra trabajando una propuesta para una nueva Ley Orgánica de Seguridad Social.

En 2005 se creó la Secretaría Nacional de los Objetivos del Milenio, con rango de Ministerio, institución que impulsó la estrategia del Aseguramiento Universal de Salud en Ecuador.

En 2006 el Banco Interamericano de Desarrollo aprobó un préstamo de 90 millones de dólares a Ecuador para apoyar el Proyecto de Aseguramiento Universal de Salud (PRO-AUS) que comenzaría por cubrir a la población más pobre del país y en su primera etapa mejoraría la calidad de los servicios de salud. En etapas subsiguientes se incorporaría a otras personas de escasos recursos y a trabajadores independientes. La experiencia arrancó en Quito y Guayaquil. Sin embargo, con el cambio de gobierno en 2007 hubo un alejamiento de la visión propuesta por el PRO-AUS y en 2008, con la definición del gobierno de impulsar otro modelo distinto al de aseguramiento, se dio por concluida esta experiencia.

La actual administración se plantea crear un sistema nacional de salud subsidiado que brindaría acceso universal a la salud sin cobro en el momento de utilización, crearía una Red Pública Integral de Salud, protegería a grupos vulnerables o con necesidades especiales, elevaría a rango constitucional las declaraciones de Doha de 2001 según las cuales el derecho al acceso a medicamentos debe anteponerse al derecho de propiedad intelectual, y fortalecería el papel rector del MSP

¿Qué impacto han tenido las innovaciones más recientes?

Posiblemente el mayor impacto de las innovaciones recientes sea la ampliación de la coordinación interinstitucional. Hasta 1998 cada institución del sector salud en Ecuador mantenía un esquema de organización, gestión y financiamiento particular sin mecanismo alguno de articulación y coordinación interinstitucional. Con la reforma de 2008, que definió el derecho a la salud, su promoción y protección, y al MSP como rector expreso, se han logrado condiciones favorables para la articulación del sector.

La inclusión de la SBS como parte de los organismos de control en el sector salud, específicamente a lo que se denominó Sistema de Seguridad Social, ha generado ya resultados interesantes que expresan la necesidad de entidades de control tipo superintendencias.

¿Cuáles son los retos y las perspectivas del sistema de salud?

Uno de los principales desafíos en materia de salud es dar cobertura a casi uno de cada cuatro ecuatorianos que actualmente no tienen acceso a ningún servicio de salud. En el gobierno anterior, después de tropiezos presupuestales y políticos, se implantó el proyecto de aseguramiento universal de salud PRO-AUS. El nuevo gobierno tiende hacia la cobertura universal para todos los ecuatorianos, para lo cual ha incrementado sustancialmente la inversión en este rubro.

Otro desafío del sector es integrar todas las instituciones en un verdadero sistema nacional de salud bajo la rectoría del MSP. Aunque muchas de las leyes y reformas constitucionales han estado encaminadas hacia ello, todavía es mucho lo que falta para concretarlo. Por ejemplo, según una disposición transitoria de la Ley de Seguridad Social, las unidades médicas del IESS tendrían un plazo de dos años para transformarse en empresas prestadoras de servicios de salud para los afiliados y la población en general. Esto no ha sucedido aunque se encuentra en camino.

Lo más relevante tal vez sea la conformación de la Red Pública Integral de Salud, que se encuentra en proceso de concertación y esperando su inicio el segundo semestre del 2011.

Declaración de conflicto de intereses: Los autores declararon no tener conflicto de intereses.

Referencias

1. Ortiz P, Albán E. Ecuador: estimaciones y proyecciones de población 1950-2025. Quito: Instituto Nacional de Estadística y Censos, 2003.
2. Instituto Nacional de Estadística y Censos. Indicadores básicos de salud. Ecuador 2009. Quito: Instituto Nacional de Estadística y Censos, 2009.
3. Ministerio de Coordinación de Desarrollo Social del Ecuador. Sistema Integrado de Indicadores Sociales del Ecuador 2010. [Accesado agosto 15, 2010]. [Consultado agosto 15, 2010]. Disponible en: http://www.siise.gob.ec/Principal.aspx.
4. Centro de Estudios de Población y Desarrollo Social. Encuesta Demográfica y de Salud Materna e Infantil. Informe final. Quito: Centro de Estudios de Población y Desarrollo Social, 2005.
5. Organización Mundial de la Salud. Ecuador. [consultado mayo 31, 2011]. Disponible en: http://www.who.int/countries/ecu/es/.
6. Organización Mundial de la Salud. Estadísticas sanitarias mundiales 2011. Ginebra: OMS, 2011.
7. Organización Mundial de la Salud. Cuentas nacionales de salud. Ecuador [consultado mayo 31, 2011]. Disponible en: http://www.who.int/nha/country/ecu/en.
8. Lozada P, Aguinaga L, Páez R, Olmedo C, Pozo A. El peso de la enfermedad en el Ecuador. Quito: CEPAR, 1999.
9. Lozada P. El peso de la enfermedad en las provincias del Ecuador. Quito: CEPAR, 2000.
10. Pan American Health Organization. Health system profile. Ecuador. Monitoring and analysis of the change and reform processes. Washington, DC: Pan American Health Organization, 2008.
11. Constitución de la República del Ecuador. Registro Oficial 2008 (20 de octubre); 449.
12. Lucio R. Modelo de salud en Ecuador. Quito: Ministerio Coordinador de Desarrollo Social, 2010.
13. Instituto Ecuatoriano de Seguridad Social. Registros institucionales 2010. Quito: Instituto Ecuatoriano de Seguridad Social, 2010.
14. Instituto de Seguridad Social de las Fuerzas Armadas. Registros institucionales 2010. Quito: ISSFA, 2010.

15. Lucio R. La salud en Quito: análisis de acceso y calidad. Quito: Cooperación Técnica Belga en Ecuador, 2007.
16. Ministerio de Coordinación de Desarrollo Social. Registros institucionales del IESS, ISSFA e ISSPOL. Quito: Ministerio de Coordinación de Desarrollo Social, 2010.
17. Ley de Maternidad Gratuita y Atención a la Infancia. Registro Oficial 1994; 9 de septiembre: 523.
18. Hermida J, Romero P, Abarca X, Vaca L, Robalino M, Vieira L. La Ley de Maternidad Gratuita y Atención a la Infancia en el Ecuador. Informe LACRSS 2005:63.
19. Decreto 347: Cámbiese el Programa Bono Solidario por Programa Bono de Desarrollo Humano. Registro Oficial 2003 (7 de mayo):763.
20. Ministerio de Inclusión Económica y Social. Programa de Protección Social 2010 [consultado agosto 15, 2010]. Disponible en: http://www.pps.gov.ec/PPS/PPS/BDH/INF/QuienesSomos.aspx.
21. Decreto ejecutivo 12: Increméntase el valor del bono de desarrollo humano a la suma de treinta dólares mensuales para las familias que se ubiquen en el primer y segundo quintiles más pobres. Registro Oficial 2007 (25 de enero): 8-10.
22. Ministerio de Inclusión Económica y Social. Red de Protección Solidaria 2010 [consultado agosto 15, 2010]. Disponible en: http://www.redsolidaria.gov.ec
23. Instituto Ecuatoriano de Seguridad Social. Seguro de Salud [consultado agosto 5, 2010]. Disponible en: http://www.iess.gov.ec/site.php?category=seguro-de-salud.
24. Ley de Seguridad Social de las Fuerzas Armadas. Registro Oficial 1992 (7 de agosto 7): 995.
25. Ley de Seguridad Social de la Policía Nacional. Registro Oficial 1995 (1° de junio 1): 707.
26. Superintendencia de Bancos y Seguros del Ecuador. Seguridad Social: Entidades Controladas [consultado agosto 5, 2010]. Disponible en: http://www.superban.gov.ec/practg/sbs_index?vp_art_id=46&vp_tip=2.
27. Ley 55: Ley de Seguridad Social. Registro Oficial 2001 (30 de noviembre 30): 465.
28. Instituto Nacional de Estadística y Censos. Encuesta de Recursos y Actividades en Salud. Quito: Instituto Nacional de Estadística y Censos, 2007.
29. Instituto Nacional de Estadística y Censos. Registro de Camas Hospitalarias. Quito: Instituto Nacional de Estadística y Censos, 2008.
30. Ministerio de Salud Pública del Ecuador. Registros institucionales. Quito: Ministerio de Salud Pública, 2010.
31. Comisión Nacional de Medicamentos e Insumos. Política Nacional de Medicamentos. Quito: Consejo Nacional de Salud, 2007.
32. Ley 2000-1: Ley de producción, importación, comercialización y expendio de medicamentos genéricos de uso humano. Registro Oficial 2005 (9 de diciembre):162.
33. Comisión Nacional de Medicamentos e Insumos. Registro Terapéutico. Quito: Consejo Nacional de Salud, 2009.
34. Comisión Nacional de Medicamentos e Insumos. Cuadro Nacional de Medicamentos Básicos. Quito: Consejo Nacional de Salud, 2009.
35. Ley Orgánica del Sistema Nacional de Salud. Registro Oficial 2002 (25 de septiembre): 670.
36. Secretaría Nacional de Ciencia y Tecnología. Rendición de cuentas [consultado agosto 15, 2010]. Disponible en: http://www.senacyt.gob.ec/?q=node/471.
37. Ley Orgánica de Transparencia y Acceso a la Información Pública. Registro Oficial 2004 (18 de mayo):337.
